# Prototyping an Ontological Framework for Cellular Senescence Mechanisms: A Homeostasis Imbalance Perspective

**DOI:** 10.1038/s41597-024-03331-y

**Published:** 2024-05-10

**Authors:** Yuki Yamagata, Tsubasa Fukuyama, Shuichi Onami, Hiroshi Masuya

**Affiliations:** 1 Laboratory for Developmental Dynamics, RIKEN Center for Biosystems Dynamics Research, 2-2-3 Minatojima-minamimachi, Chuo-ku, Kobe, Hyogo 650-0047 Japan; 2 Life Science Data Sharing Unit, Infrastructure Research and Development Division, RIKEN Information R&D and Strategy Headquarters, 2-2-3 Minatojima-Minamimachi, Chuo-ku, Kobe, Hyogo 650-0047 Japan; 3 AXIOHELIX CO. LTD., 8F Kubota Bldg., 1-12-17 Kandaizumicho, Chiyoda-ku, Tokyo, 101-0024 Japan; 4 Integrated Bioresource Information Division, RIKEN BioResource Research Center, Kouyadai 3-1-1 Tsukuba, Ibaraki, 305-0074 Japan

**Keywords:** Gene ontology, Senescence

## Abstract

Although cellular senescence is a key factor in organismal aging, with both positive and negative effects on individuals, its mechanisms remain largely unknown. Thus, integrating knowledge is essential to explain how cellular senescence manifests in tissue damage and age-related diseases. Here, we propose an ontological model that organizes knowledge of cellular senescence in a computer-readable form. We manually annotated and defined cellular senescence processes, molecules, anatomical structures, phenotypes, and other entities based on the Homeostasis Imbalance Process ontology (HOIP). We described the mechanisms as causal relationships of processes and modelled a homeostatic imbalance between stress and stress response in cellular senescence for a unified framework. HOIP was assessed formally, and the relationships between cellular senescence and diseases were inferred for higher-order knowledge processing. We visualized cellular senescence processes to support knowledge utilization. Our study provides a knowledge base to help elucidate mechanisms linking cellular and organismal aging.

## Introduction

Multicellular organisms are products of repeated rounds of cell growth and division^1^. After repeated divisions, several cells stop proliferating. This phenomenon is called cellular senescence and represents a cell fate characterized by stable proliferative arrest in response to various stressors^2^. It is implicated as a fundamental contributor to aging and chronic disease^2^. Consequently, the use of senotherapy or senolytics to ameliorate aging by eliminating senescent cells has become a research focus^3,4^.

Cell states link both physiological and stress signals to tissue homeostasis and organismal health^5^. Both organismal aging and cellular senescence are associated with the disturbance of homeostasis. Aging has been considered the collapse of homeostasis^6^, and cellular senescence is seen as a process that maintains cell viability when a cell can no longer contribute to continued homeostasis via cell division^7^.

Cellular senescence presents both intrinsic advantages and disadvantages for organisms^3^; however, its mechanisms remain largely unknown. Organisms are comprised of complex, heterogeneous, and granular structures with various functions. To understand the aging-related phenotypes of organisms, it is necessary to explain the role of cellular senescence in tissue damage, pathological changes, and aging-related diseases at levels ranging from cellular to organismal. Clarifying these mechanisms will require expertise in multiple fields, including molecular biology, cell biology, pathology, pharmacology, clinical medicine, and gerontology. Hence, aging research would benefit from interdisciplinary knowledge of the mechanisms of cellular senescence.

Various ontologies have been developed for biomedical research. Notably, Gene Ontology (GO) has played a major role in providing terms in a machine-readable manner and has contributed to the functional analysis of genes and sharing of knowledge^8^. Developing an ontology that can describe and organize entities related to cellular senescence would significantly promote studies on aging by permitting the integration of relevant data and knowledge. However, a comprehensive overview of cellular senescence—from aberrant molecular signaling to disturbances in biological processes and even disease progression—demands cross-domain integration of multiple ontologies. The OBO Foundry attempts to describe domain ontologies in a unified framework based on an upper ontology, Basic Formal Ontology (BFO)^9^. Within the initiative, we developed TXPO^10^ and subsequently extended it, named Homeostasis Imbalance Process ontology (HoIP)^11^. One of the advantages of HoIP is that it defines various domain-independent functional processes and can be used to define anomalies in the functions based on a “homeostasis imbalance” perspective. HoIP is characterized by high consistency through manual curation and offers extensibility. It also supports the application of ontology terms using the Web Ontology Language - Description Logic (OWL-DL)^12^.

Considering such advantages of HoIP, we decided to develop a value-added ontology that extends HoIP to integrate and organize basic knowledge related to cellular senescence to support aging research, according to the following systematic approach.

### Description of the high-level knowledge that is considered a value-added finding

We focused on the intricate process of cellular senescence and its contribution to chronic conditions such as type 2 diabetes. This high-level knowledge, bridging molecular, cellular, and organ systems, offers profound insights into disease mechanisms. By focusing on homeostasis imbalance and elucidating the progression of cellular-level processes leading to systemic diseases, —diverging from conventional approaches in the domain — we aimed to contribute novel perspectives for developing early-stage interventions to prevent or mitigate chronic illnesses in aging populations.

### Provenance of the basic knowledge that was processed to enable the high-level knowledge

The foundational knowledge for our study was sourced from geriatric medicine^6^, pathology^7^, and biology of aging textbooks^13^, and review articles selected considering their comprehensive coverage of the field. The approach ensured a rich interdisciplinary understanding of cellular senescence and aging, from molecular biology to clinical implications, providing a solid base for our high-level findings.

### Method/algorithm yielded the added value

Dispersed and diverse knowledge from textbooks and reviews was integrated into a unified ontological representation framework using the HoIP. OWL-DL allowed for the consistent and coherent representation of knowledge across different granularities. Additionally, using tools such as Cytoscape to visualize causal relationships in cellular senescence mechanisms enhances understanding and facilitates further discovery within the field.

### Differentiation from other methodologies represented by large-scale language models

Large-scale language models (LLMs) and knowledge graphs offer powerful tools for knowledge discovery; however, they have some limitations. LLMs, for instance, may suffer from ‘hallucinations’ and lack logical consistency. Concerning knowledge graphs, integrating a wide array of terms without a coherent framework could potentially lead to inconsistencies. Our ontological framework provides a structured, logical foundation for exploring and expanding on complex biological phenomena. The approach effectively provides consistent representation for knowledge graphs. To achieve the objectives, we focused on systematizing knowledge by developing the HoIP ontology. Previously, the HoIP ontology organized knowledge regarding the causal relationships between biological processes and processes triggered by external factors, such as toxic mechanisms and coronavirus disease 2019 (COVID-19). In the present study, we have also extended the ontology to include intrinsic factors, specifically aiming to systematize knowledge about the mechanisms involved in cellular senescence. Based on these perspectives, we designed the ontology to serve as a knowledge base with a capacity for higher-order knowledge processing. Although this study presents a prototype, focusing primarily on the underlying fundamental mechanisms, the ontology, the Cellular Senescence Expanded HoIP, could facilitate novel insights into cellular senescence, enhancing our understanding of cellular senescence and aging, in addition to treatment of age-related diseases.

## Results

### Ontology development based on HOIP for organizing cellular senescence knowledge

**Fig. 1:**
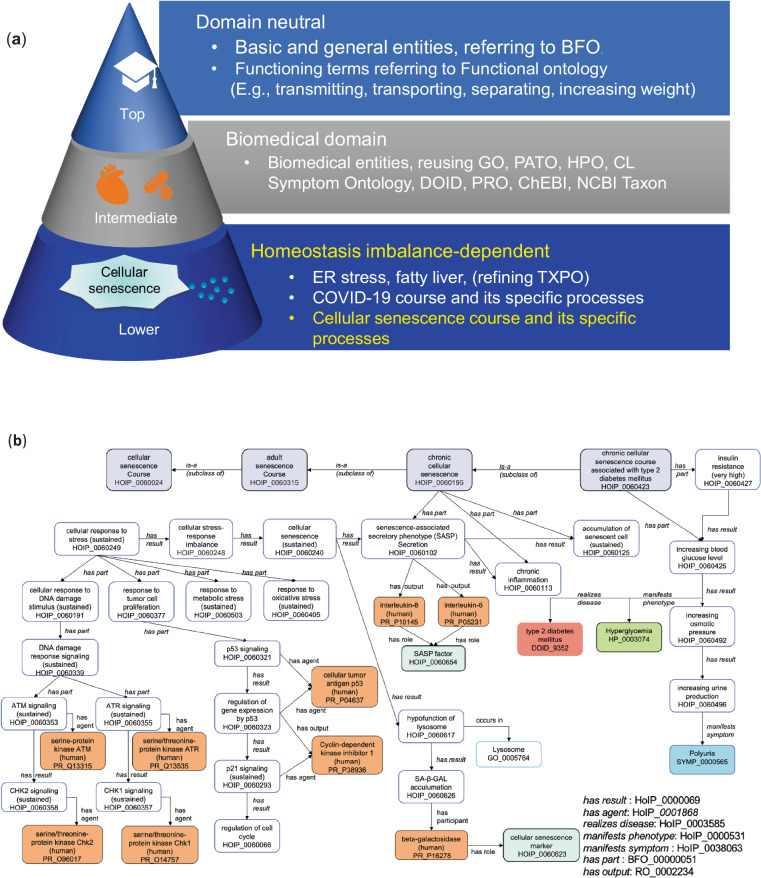
Schematic representation of cellular senescence and HOIP ontology. (**a**) Overview of the organization of homeostasis imbalance process ontology (HOIP). The HOIP ontology comprises three layers. The top layer is domain-independent and defines basic and general terms that refer to the BFO and functional terms that can be used across domains. The intermediate layer is biomedicine-dependent and reuses the OBO Foundry ontology, such as GO, CLO, HPO, DOID, and Symptom Ontology. The lower layer depends on homeostasis imbalance and defines cellular senescence processes and courses. (**b**) Ontological representation pattern of the cellular senescence course in HOIP. Cellular senescence courses are shown in gray. The molecules are indicated in orange, role in light green, phenotype in green, symptom in blue, and disease in red. Arrows indicate relationships.

To systematically describe the homeostatic disturbance processes underpinning cellular senescence, we utilized the HOIP as the foundation for knowledge modelling. HOIP consists of three layers and has some fundamental advantages (Fig. 1a). HOIP refers to the top-level BFO^9^ commonly used in over 100 ontology projects worldwide, defining terms with a domain-independent objective viewpoint at the highest layer. HOIP also defines domain-independent functional processes based on functional ontology^14^, which allows specialized definitions of biological functional processes as subclasses acting to maintain homeostasis. In the intermediate layer, HOIP defines the biological processes essential for maintaining homeostasis. To permit interoperability of the knowledge, terms widely used in a biomedical ontology in the OBO Foundry^15^, such as GO, the phenotype ontologies Phenotype And Trait Ontology (PATO)^16^ and Human Phenotype Ontology (HPO)^17^, the Uber-anatomy ontology (UBERON)^18^, the cell ontology (CL)^19^, the Symptom Ontology^20^, the disease ontology (DOID)^21^, the protein ontology (PRO)^22^, Chemical Entities of Biological Interest (ChEBI)^23^, and the National Center for Biotechnology Information (NCBI) taxonomy^24^, were imported into the intermediate layer of the HOIP. The bottom layer relates to the homeostatic imbalance. Here, to deal with the mechanism of homeostasis disturbances, HOIP defines a “homeostatic imbalance course,” which is a process sequence consisting of multiple processes as a subclass of the BFO’s “process.” We adopted the GO definition for cellular senescence: “A cell aging process stimulated in response to cellular stress, whereby normal cells lose the ability to divide through irreversible cell cycle arrest.” This aligns with the concept of “cell fate characterized by stable proliferative arrest in response to various stressors^2^.” Using the definition in our HoIP ontology, we first annotated stressors, then their responses—such as DNA damage response (DDR)—leading to proliferative arrest, cellular senescence, and the outcome. The approach enabled us to systematically annotate processes from the cellular to the organismal levels, constructing a coherent ontology that objectively captures the components of complex biological phenomena.

Our approach to relationships is grounded in the principle of reusability of the Relations Ontology (RO)^25^. Concerning the ‘has output’ relationship, we have utilized the corresponding relation from RO (RO:0002334) to ensure consistency with established biomedical ontologies. However, the creation of the ‘has result’ relationship was necessitated by the unique requirements of our study. RO provides ‘causally upstream of’ (RO:0002418), which is the subclass of ‘causal relation between processes’ (RO:0002411) and the subclass of ‘precedes’ (BFO:0000063) as a temporal sequence. Considering the complexities associated with aging, particularly concerning life stages and cellular timing, we defined a new, specific relationship within the HoIP ontology to more precisely represent these causal relationships without the inherent temporal assumptions.

Regarding the properties related to diseases, while ‘contributes to severity of condition’ (RO:0003305) from RO was considered, it lacks domain and range restrictions, allowing for broad applicability. In contrast, our ontology aims to delineate processes as the domain and diseases as the range, to clearly differentiate between phenotypes and diseases. To achieve such specificity and clarity, new relationships tailored to our ontology were established. By introducing such novel relationships, the current approach facilitates more precise and meaningful annotations within the domain of cellular senescence and its implications for diseases.

HOIP is described by OWL-DL language^12^ for both human and machine readability. Thus, we maximized the advantages of HOIP and defined the domain knowledge of cellular senescence in the bottom layer. To provide domain-specific knowledge of cellular senescence at the homeostatic imbalance bottom layer, we newly defined terms describing stress responses that act to maintain homeostasis as the functional process in the cellular senescence mechanism (Fig. 1 and Table S1). We also defined terms relating to the stresses as functional demands. By challenging the understanding of the causes of processes, we described the mechanism in a unified representation framework as causal relationships and introduced a homeostasis imbalance model. This model illustrates how homeostasis disturbance could lead to tissue damage via cellular senescence, with applications to organismal aging and diseases.

### Definition and types of cellular senescence courses to elucidate mechanisms

We first established what processes may occur during cellular senescence. We defined the cellular senescence course, a subclass of “process sequence,” as “the totality of all processes through which cellular senescence is realized.” Subsequently, we classified cellular senescence courses into embryonic and adult cellular senescence courses. In the adult cellular senescence course, as the accumulation of senescent cells can induce pathological processes, we further classified subclasses and defined acute and chronic cellular senescence mechanisms^26^.

**Cellular senescence course (HOIP_0060024)**: A course constituting multiple processes that lead to cellular senescence.

**Embryonic cellular senescence course (HOIP_00600267)**: A cellular senescence course that occurs during embryogenesis.

**Adult cellular senescence course (HOIP_0060315)**: A cellular senescence course in adults.

**Acute cellular senescence course (HOIP_0060196)**: An adult cellular senescence course that can cause transient cellular senescence.

**Chronic cellular senescence course (HOIP_0060195)**: An adult cellular senescence course that can result in a sustained cellular senescence process, which may lead to the senescence-associated secretory phenotype (SASP), and chronic inflammation due to senescent cell accumulation.

In addition, we defined chronic cellular senescence associated with type 2 diabetes mellitus (HOIP_0060423) because it is related to chronic diseases in advanced age as described in a review article^26^. (Fig. 1b).

### Computational representation of cellular senescence knowledge

1. Annotated information for human experts
  HOIP is based on OWL, which enables reasoning by description logic. However, it is difficult for domain experts to understand the content. Therefore, in line with the HOIP development policy, information for human domain experts was provided using OWL annotation properties. All definitions of entities involved in cellular senescence were newly described in natural language. In addition, entities were manually annotated from textbooks, reviews, and journal articles by human experts. Moreover, to enable researchers to follow the evidence trail behind annotation of each cellular senescence-related process, reference information (PubMed ID [PMID]) was assigned (Fig. 2a) as an external cross-reference. The information is available on the BioPortal site, for instance, under the process ‘CHK1 signaling [adult cellular senescence]’, and the PMID:33328614 is displayed as a database cross-reference at the URL (http://purl.bioontology.org/ontology/HOIP/HOIP0060144), allowing users to access the article related to each process. Moreover, we have recently launched a knowledge exploration system based on the HoIP ontology, named Leaves (https://leaves.riken.jp/)^27^, where each process node in the course displays ontology information, including article information (Fig. 2b).

**Fig. 2:**
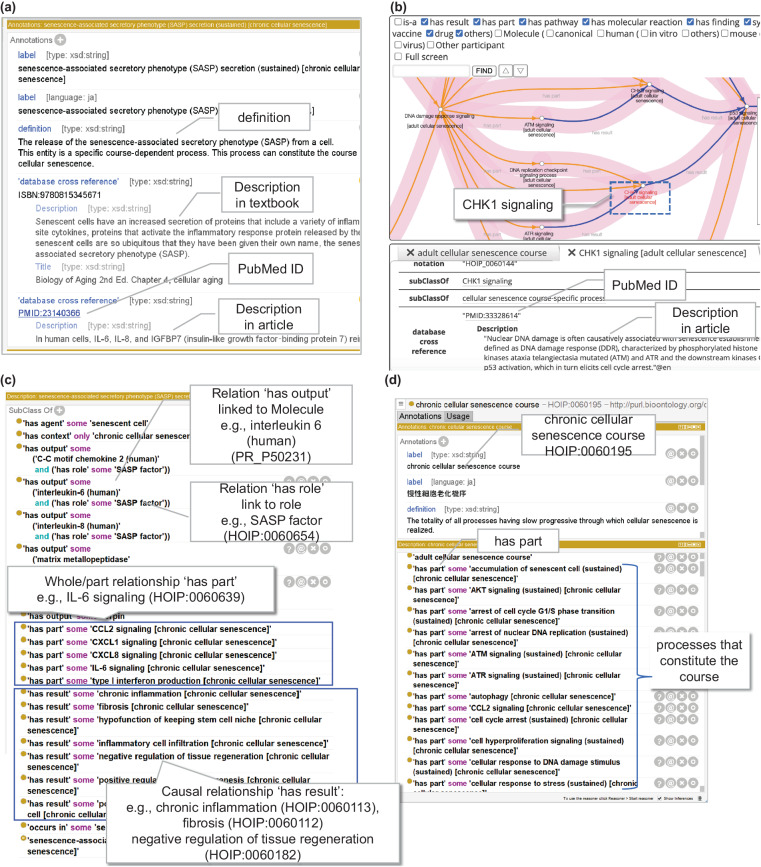
Examples of computational representation of senescence-associated secretory phenotype (SASP) secretion (HOIP:0060102) and chronic cellular senescence course (HOIP:0060195) with Protégé software. (**a**) A screenshot from Protégé for an example of a description using the annotation properties. Descriptions by natural language are given for the “definition” and “Description” from textbooks or articles, and links to external references with “PubMed ID” are shown by balloons. (**b**) Example of PubMed article information in the Process tab of Leaves using HoIP. (**c**) Example of a description using object properties of the process. The major entities involved in the process are indicated by balloons. In this example, relations to process as senescence-associated secretory phenotype (SASP) secretion, (1) whole-part (‘has part’) relation linked to sub-process as IL6 signaling, (2) causal relation (‘has result) linked to chronic inflammation, (3) relation (‘has output’) linked to a molecule such as interleukin-6 (human), and (4) relation ‘has role’ linked to a role such as SASP factor are shown. (**d**) The processes constituting the courses are shown in square surroundings described by ‘has part’ relationships (balloon).
  We aimed to elucidate general mechanisms of cellular senescence, as well as their implications in clinical symptoms and diseases specific to humans. Therefore, in the present study, annotations of processes are based on humans, referring to textbooks of not only biology but also gerontology, pathology, and internal medicine and review articles related to humans (see Methods). Molecular entities involved in cellular senescence processes were annotated manually, referred to as the Protein Ontology, and we added cross-references to the HUGO Human Gene Nomenclature Committee (HGNC)^28^. For processes commonly described among mammals in textbooks and review articles, the annotation of orthologous mouse genes/molecules from the Protein Ontology was included in the ontology. It should be noted here that data using the annotation properties are supplementary information for the machine and do not affect the inference. In other words, our descriptions distinguished cellular senescence knowledge in a human-readable form for human experts from that used for computer processing for ontological reasoning.
2. Description and formalization for computer processing

1. Cellular senescence-specific processes and related entities
  HoIP ontology is described in the OWL-DL format, leveraging its compatibility with description logic principles. Description Logic (DL) syntax was used, with constructors such as subsumption (⊆), intersection (⋂), universal restriction (∀), and existential restriction (∃). The constructors correspond to logical operators in first-order logic, facilitating the structured representation of complex relationships within our ontology. The use of the syntax allows ontology reasoning tools to effectively check for consistency across the defined classes and relationships.
  The SASP secretion (HOIP:0060102) process can be expressed using the logical equation provided in Supplementary Information 1.
  We described the characteristics of each entity and its relationships with other entities using object properties in OWL. Parent classes were also identified. Figure 2c depicts a representation of the entity SASP secretion (HOIP:0060102) newly defined in the HOIP using OWL object properties by Protégé software as an example of a process specific to cellular senescence. A process can have sub-processes; for example, SASP secretion has interleukin (IL)-6 signaling (HOIP:0060639) as a sub-process as determined using the object property ‘has part.’ Furthermore, if a process could lead to another process, we described the causal relationship (using the ‘has result’ relation). Figure 2c depicts chronic inflammation (HOIP:0060113) due to sustained SASP secretion. In the case of SASP secretion, besides chronic inflammation, it can lead to multiple results, such as fibrosis (HOIP:0060112) and negative regulation of tissue regeneration (HOIP:0060182). According to one paper^26^, SASP-mediated extracellular matrix remodelling may play a key role in disease progression and suppression, and senescent cells promote lung fibrosis but suppress liver fibrosis.
  We used the ‘occurs in’ relationship to describe the location of the process. For example, SASP secretion occurs in senescent cells. In the SASP secretory process, interleukin-6 and interleukin-8 are secreted and represented using the ‘has output’ relation. We identified the role of each molecule in processes as context, such as the ‘SASP factor’ role, using the ‘has role’ property. Furthermore, as symptoms are essential in clinical medicine, the ‘manifests symptom’ relationship was used. For example, the process ‘increasing urine production (HOIP:0060496)’ manifests as ‘polyuria (SYMP_0000565).’ In a case where a process manifested a disease, the ‘realizes disease’ relationship was used to describe diseases, such as type 2 diabetes mellitus (DOID:9352) (Fig. 1b). With these properties, we could infer related symptoms and diseases using DL-query.
2. Cellular senescence courses

Figure 2d depicts an example of a representation of the chronic cellular senescence course (HOIP: 0060195) using Protégé. The processes defined above (1) were enumerated using the ‘part of’/‘has part’ relation as the object property for each course of cellular senescence, and they were represented as belonging to the course, that is, as constituting a course. The subclasses of the course inherited the processes of the course of the superclass, specialized them, or added new processes. For example, cellular senescence (HOIP:0060129) in the cellular senescence course was specialized to cellular senescence (sustained) (HOIP:0060240) in the chronic senescence course, and processes such as accumulation of senescent cells (sustained) (HOIP:0060308) and chronic inflammation (HOIP:0060113) were added to the components. This equation is presented in Supplementary Information 2.

3. Unified representation model of homeostatic imbalance in cellular senescence: causal networks in cellular senescence mechanisms

**Fig. 3:**
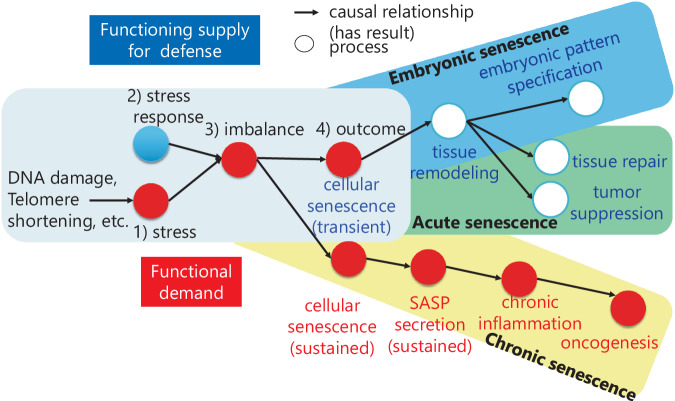
A unified representation of cellular senescence courses. An overview of the unified representation of cellular senescence courses. The blue terms indicate processes that can be caused by transient cellular senescence, and the red terms indicate processes that can be caused by sustained cellular senescence. The squares indicate the basic components of homeostatic imbalance: (1) stress, (2) stress response, (3) imbalance, and (4) outcome. The embryonic cellular senescence course is shown in a blue round box, the acute cellular senescence course is shown in a green round box, and the chronic cellular senescence course is shown in a yellow round box.

Because each process in the cellular senescence course has a causal relationship, a network (causal network) consisting of linked causal relationships among multiple processes was generated by linking them computationally. Here, we applied the imbalance balance model to the network and provided a unified description by representing the imbalance between functional demand and the functioning process^10,29^. As a result of the formalization described above, a unified representation framework model was constructed to describe the progression of the cellular senescence mechanism as follows: (1) stress (functional demand process), (2) stress response as the functioning process to maintain homeostasis, (3) homeostatic imbalance, and (4) outcome, which is cellular senescence, as summarized in Fig. 3.

Using the OntoGraph Application Programming Interface (API) of Protégé, our model could represent that in the embryonic cellular senescence course and acute cellular senescence courses, the homeostatic imbalance could lead to transient cellular senescence, as shown in Fig. S1a and b. In embryonic cellular senescence, our model showed an imbalance in stress as an increasing demand for pattern specification, and the response could result in transient cellular senescence. Consequently, tissue remodelling can result in embryonic pattern specification (Fig. 3, Fig. S1a). In the case of acute cellular senescence, our model described that as part of the increasing demand for a cellular stress response, there might be an increasing demand for the oncogenic stress response, which has a further subprocess, PTEN loss. Furthermore, the imbalance between stress and its response as a defence function could lead to transient cellular senescence, which might lead to negative regulation of tumour proliferation (Fig. S1b). In contrast, in chronic cellular senescence, according to the imbalance model, persistent cellular senescence, leading to SASP secretion, could cause further chronic inflammation (Fig. S1c).

In summary, our ontology and the imbalance model enable a unified representation of the causal networks of cellular senescence.

### Evaluation of ontology

For evaluation of the constructed ontology, we introduce the two terms ontology verification and validation^30,31^. Ontology verification refers to building the ontology correctly, which pertains primarily to consistency and does not extend to comprehensiveness. We used the ontology reasoner HermiT^32^ and ELK^33^ in Protégé for formal verification. The results showed no violations of the domain or range of the property and class hierarchy, and there was consistency in terms of formality. Next, ontology validation refers to assessing the extent to which the ontology definitions accurately represent the real world for which the ontology was created^30,31^. Our study focused on applying this principle to the causal relationships involved in cellular senescence. By applying transitivity in ontology reasoning, we aimed to validate that the content of our ontology reflects the descriptions in selected articles and textbooks on cellular senescence. The approach was intended to meet the requirements of users interested in comprehending or investigating the complex cellular senescence phenomena.

1. Validation of the causal network of p21 signaling
  The mechanism of cellular senescence involves p16 and p21 signaling^3,26,34^. Therefore, we validated the causes of p21 signaling using ontology reasoning as a case study. We used a transitive property, owl:Transitive Property, for the causal relation (‘has result’), which infers not only direct causal relations but also indirect causal relations. According to the results, in adult cellular senescence (HOIP:0060315), for example, p21 signaling (HOIP:0060325) could occur via ATM signaling (HOIP:0060110), p53 signaling (HOIP:0060055), and regulation of gene expression by p53 (Fig. S2a). In contrast, in the embryonic senescence course (HOIP:0060267), the findings revealed that p21 signaling could occur via TGF beta signaling (HOIP:0060269), SMAD signaling (HOIP:0060290), and regulation of gene expression by SMAD (Fig. S2b). Thus, the differences among the courses can be represented by different paths in the causal networks.
2. Validation of causal network including part of the whole relation

Next, we assessed whether the causes were clearly described, including the relationship between the whole process and its parts. We examined the causes of p21 signaling in the adult cellular senescence course, including part-whole processes.

We used the OWL2 property chain axiom and defined a new property, “has part result,” to connect processes linked by a chain of two relationships: the “has part” and “has result” properties. The approach aids in inferring more complex relationships between whole-part processes and causal relationships, which are not described explicitly. In the context of the stress response, the signaling response is inferred, with sub-pathways integral to the overall response. Consequently, ATR signaling (HOIP:0060140) and ATM signaling (HOIP:0060110) are inferred as upstream of p21 signaling (HOIP:0060325), as sub-processes of the DDR signaling (HOIP:0060337) (Fig. S2c–e). The results are further supported by the assertion^34^ that ‘they activate the DNA damage response (DDR), a signaling pathway in which ATM or ATR kinases block cell-cycle progression through stabilization of p53 and transcriptional activation of the cyclin-dependent kinase (Cdk) inhibitor p21’.

Thus, the present study infers that our description can explain how the processes involved in cellular senescence, including the whole-part relationships, can be affected.

We also confirmed that SPARQL Protocol and RDF Query Language (SPARQL) queries (Supplementary Information 3) could acquire the processes of chronic cellular senescence (HOIP:0060195), including telomere shortening, p16 signaling (HOIP:0060294), p53 signaling (HOIP:0060321), and senescence-associated heterochromatin focus formation (HOIP:0060162).

### Causal inference of disease-related processes

As a case study, we used our ontology to infer the possible causal relationships between the cellular senescence process and type 2 diabetes mellitus based on a DL query.

1. Exploring the relationship between cellular senescence and type 2 diabetes mellitus
  The relationship between cellular senescence and diseases remains a compelling question. Ontology-based inference is expected to support the discovery of causal networks between cellular senescence and diseases through computational processing. In the present study, manual annotation was carried out by human annotators with expertise in the biomedical sciences. The annotators were tasked with identifying descriptions of causal relationships between processes related to type 2 diabetes mellitus within each reference. The work is crucial for accumulation of the domain expert knowledge entered into our ontology. Subsequently, ontologists utilized the Protégé tool to formalize the descriptions in the OWL-DL language, enabling a structured representation of the knowledge. The step is essential for facilitating ontological reasoning, which is at the core of our methodology. With the structured knowledge representation in OWL, we utilized ontological reasoner ELK to automatically derive indirect causal relationships across multiple references. This was achieved through the logic using transitivity of OWL that allows for the discovery of latent paths between cellular senescence and type 2 diabetes mellitus.
  Figure 4a depicts a list of processes involved in the causes of insulin resistance (HOIP:0060427). One hypothesis was that telomere shortening (HOIP:0060431) could cause type B pancreatic cell exhaustion/hypofunction of type B pancreatic cells (HOIP:0060453), resulting in positive regulation of SASP secretion (HOIP:0060455) and SASP secretion (HOIP:0060432), which might lead to insulin resistance (HOIP:0060427)^26^. Other processes included FOXO signaling (HOIP:0060504) and TLR4 signaling (HOIP:0060512) (Fig. 4b)^35^.

**Fig. 4:**
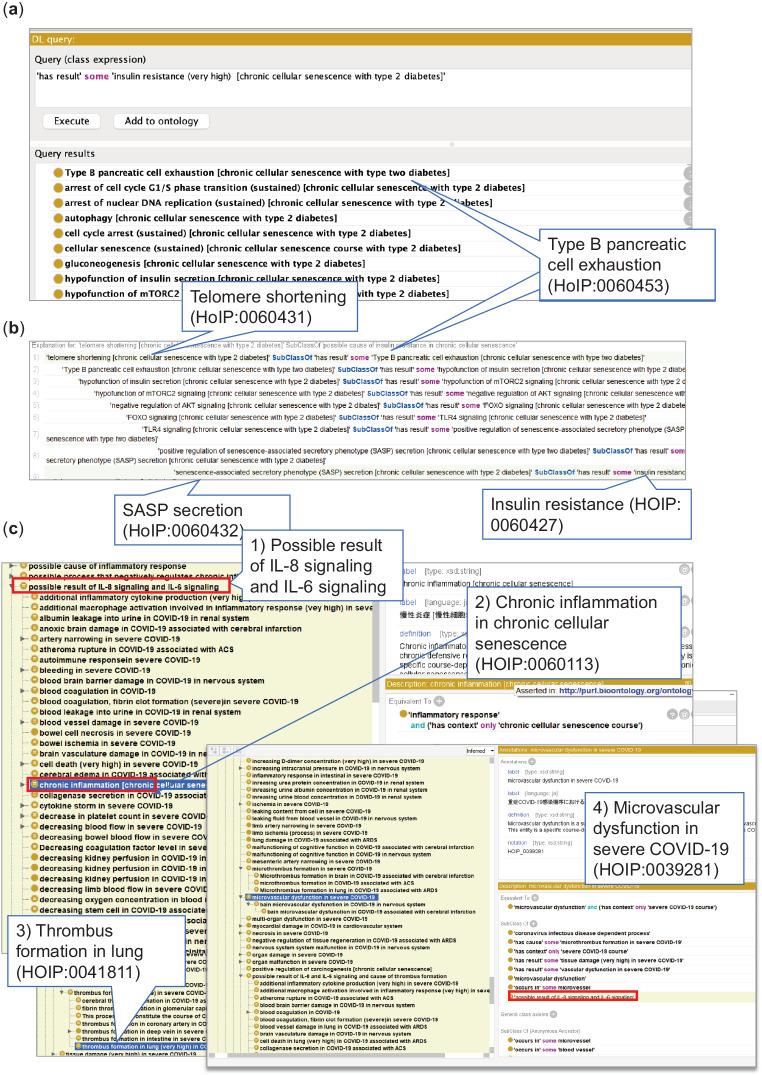
Example of causal inference relevant to diseases in the Protégé software. (**a**) Inference results in DL query. Upper panel shows the query for the search of causal relationships (has result) of insulin resistance (HOIP:0060427) in chronic cellular senescence associated with type 2 diabetes mellitus (HOIP:0060423). The lower results show the possible cause of the processes by computational reasoning, for example, Type B pancreatic cell exhaustion (HOIP:0060453 (shown in the balloon)). (**b**) Inference shows that telomere shortening (HOIP:0060431), indicated in the balloon, might cause insulin resistance (HOIP:0060427) via positive regulation of senescence-associated secretory phenotype (SASP) (HOIP:0060455) and senescence-associated secretory phenotype (SASP) secretion (HOIP:0060432) (balloon). (**c**) Results of causal inference of IL-6 signaling (HOIP:0004327) and CXCL8 (IL-8) signaling (HOIP:0041903) across courses used in the ELK reasoner in Protégé. The upper red frame shows (1) the possible result of IL-6 signaling (HOIP:004327) and CXCL8 signaling (HOIP:0041903) across courses for the validation of causal networks. Inferred information is shown in a yellow background, and (2) chronic inflammation in chronic cellular senescence (HOIP:0060113) and (3) thrombus formation in the lung (very high) in COVID-19 associated with ARDS (HOIP:0041811), and (4) microvascular dysfunction in severe COVID-19 (HOIP:0039281) is inferred as the possible result of IL-8 and IL-6 signaling. The red frame indicates that the microvascular dysfunction term is classified as a possible result of IL-8 and IL-6 signaling by inference.
2. SASP factors and cross-mechanism causal search: relationship to COVID-19 severity mechanisms

During chronic cellular senescence, the accumulation of senescent cells can cause SASP secretion and lead to IL-6 signaling (HOIP:0060639) and CXCL8/IL-8 signaling (HOIP:0060640). Therefore, we inferred the possible effects of IL-6 and IL-8 signaling across courses in HOIP. Our analysis inferred a significant overlap of inflammation-related processes between cellular senescence course and COVID-19, previously reported separately^36,37^. Moreover, the inference predicted that the possible effects of IL-8 and IL-6 signaling included processes associated with severe COVID-19, such as microvascular dysfunction (HOIP:0039281) and thrombus formation in the lung (HOIP:0041811) (Fig. 4c).

### Visualization of the cellular senescence causal network

**Fig. 5:**
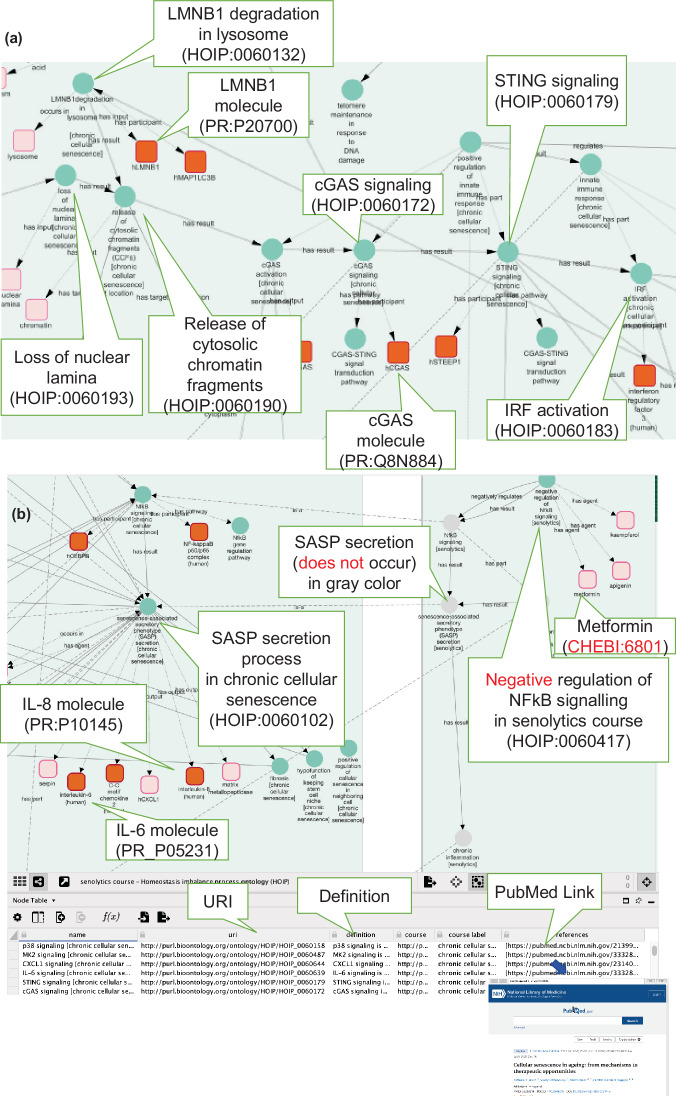
Cytoscape visualization of the cellular senescence course. The graph visualizes causal networks between processes and molecules involved in cellular senescence. Nodes denoted with circles indicate processes, and squares indicate material entities. The red color indicates molecules in humans. Balloons show explanations of visualized components. (**a**) Part of a screenshot visualizing the chronic cellular senescence course from LMNB1 degradation to IRF activation using Cytoscape. In the process of LMNB1 degradation in lysosomes (HOIP:0060132), the molecule LMNB1 (PR:P20700) (red color) is visualized. The downstream visualization showed that the degradation of LMNB1 could cause the release of cytosolic chromatin fragments (CCFs) (HOIP:0060190) (balloon), leading to cGAS signaling (HOIP:0060172) and STING signaling (HOIP:0060179), which might result in IRF activation (HOIP:0060183). (**b**) Part of the screenshot to visualize the process inherited from the super-class which is represented by the “is-a” edge (dot line) across super-class and sub-class courses. In the senolytics course (HOIP:0060418), NFKB signaling in chronic cellular senescence is inherited, and negative regulation of NFKB signaling (HOIP:0060417) by drugs such as metformin (CHEBI_6801) in the senolytics course could cause no NFKB signaling. As a result, downstream, no SASP secretion might occur (right side, gray node). In the lower table, information from the ontology, such as the definition, and a PubMed link are shown.

The visualization of ontologies is expected to have various applications. Ontologies are often published as knowledge graphs in Resource Description Framework (RDF) format, where a SPARQL query retrieves the necessary data against the SPARQL endpoint, which is a database that stores RDF data. However, it is difficult for senescence specialists to handle complex SPARQL queries. Therefore, in this study, we visualized cellular senescence mechanisms using Cytoscape^38^ so that specialists in aging who are unfamiliar with ontologies and SPARQL queries could easily use cellular senescence knowledge in ontologies. This study used SPARQL queries as the background process. The relationships were visualized in an easy-to-understand manner by converting the query results into nodes and edges of a graph using the CyREST API (Fig. 5, see Methods and Fig. S3).

Our visualization model allows users to search for molecules and processes. We confirmed that Cytoscape could create subgraphs of cellular senescence by simply selecting the types of edges by filtering and could also extract and visualize the neighbouring nodes of processes and molecules of interest. In addition, tables can access ontology information in ontology repository sites such as BioPortal^39^ from the URI information of each node. In HoIP, several processes were defined as a sub-class of GO, and the GO URI enabled access to the external GO site from Cytoscape. For each process, via external links to PubMed, a literature search of PubMed was displayed. An example of a part of the visualization of the chronic cellular senescence course is shown in Fig. 5. In the process LMNB1 degradation in lysosome (HOIP:0060132), LMNB1 (PR_P20700) could be visualized. Downstream analysis inferred that degradation could cause the release of cytosolic chromatin fragments (CCFs) (HOIP:0060190), leading to IRF activation (HOIP:0060183) via cGAS-STING signaling (cGAS signaling [HOIP:0060172], STING signaling [HOIP:0060179]), as an innate immune response (Fig. 5a)^5^.

For visualization, the process inherited from the super-class was represented by the “is-a” edge across the super-class and sub-class courses. For example, NFKB signaling in chronic cellular senescence is inherited, and negative regulation of NFKB signaling (HOIP:0060417) by drugs such as metformin (CHEBI_6801) in the senolytics course (HOIP:0060418) could inhibit NFKB signaling. Consequently, no SASP secretion might occur downstream (Fig. 5b, right side, grey node).

Thus, we demonstrated that information on cellular senescence was presented graphically, with the nodes corresponding to processes and molecules. Other information about cellular senescence in HoIP is presented in tables, allowing the user to navigate literature and ontologies.

We made the visualization available on Network Data Exchange (NDEx)^40–46^ for easy web access because users would otherwise need to install Cytoscape (https://www.ndexbio.org).

## Discussion

In this study, we developed an ontology to establish a knowledge base focused on cellular senescence. We systematized a wide array of knowledge relating to cellular senescence dispersed in textbooks and articles from multiple domains into a consistent form that can be processed using computers. By applying HoIP as the fundamental tool for knowledge management, we expect to contribute to both theoretical and practical aspects such as senolytics and senotherapy in drug development. As major biomedical ontologies such as GO, UBERON, HPO, and ChEBI have been referred to in HoIP, the ontology will accelerate interoperability and further accumulation of knowledge.

Knowledge representation based on a unified description by the homeostasis imbalance model enabled us to explain some differences and commonalities among cellular mechanisms.

Quantifying “imbalance” is considerably challenging. Taking diabetes as an example, an imbalance where the demand for insulin (functional requirement) exceeds its supply leads to hyperglycaemia. The outcome facilitates the differentiation between type 1 and type 2 diabetes. Type 1 is marked by absolute insulin deficiency, often leading to persistent hyperglycaemia, which serves as a key indicator of the condition. Similarly, in cellular senescence, the distinction between chronic senescence and embryonic/acute senescence is made based on whether the accumulation of senescent cells is sustained. This is determined by the imbalance between stress response and stress response demand, resulting in the persistent presence of senescence cells. HoIP ontology acknowledges the complexity of identifying a clear threshold for “imbalance,” while providing a representation framework for understanding the dynamics of cellular senescence.

Our model described that the common outcome of embryonic and acute cellular mechanisms is the transient occurrence of cellular senescence, which results in life-supporting activities, as shown in Fig. S1a and b. With regard to the stress exerted on the embryo, this study considers the demand for dynamic organogenesis and embryonic pattern specification as endogenous stressors that cause a disruption in local static homeostasis. Such disruption may lead to transient cellular senescence, potentially causing tissue remodelling and pattern formation during embryonic development (Fig. S1a). Our model suggests that this dynamic interplay between stress and cellular response may underlie the observed cellular senescence rather than the conventional static view of homeostasis maintenance in adult tissues. Although homeostasis is a static state, disturbance of homeostasis might induce so-called homeorhesis, as proposed by Waddington^47^, which leads to a dynamic equilibrium state. In the case of acute cellular senescence, the imbalance between the stress of activation of oncogenes and defence responses could lead to transient cellular senescence as well as embryonic cellular senescence, resulting in negative regulation of tumour proliferation (Fig. S1b). Thus, our ontology represented that embryonic and acute cellular senescence might sustain life by inducing tissue stability while providing dynamism at the cell level via temporary, local cellular senescence.

In contrast, our analysis indicates that the sequence of events in chronic cellular senescence, which includes chronic inflammation, tissue damage, and the positive regulation of carcinogenesis, suggests a pathway to organ malfunction in the organism. Thus, our model reflects biological phenomena in a computational processable way, indicating that different types of cellular senescence can be beneficial or detrimental to life.

One of the difficulties in sharing knowledge is that each expert has a different knowledge level depending on his/her background. Ontology can solve this problem by providing both general and domain-specific knowledge. The hierarchical ontology structure helps identify commonalities and differences among the diversity of expert knowledge. Furthermore, the ontology can be verified using DL. In this study, the description of HoIP is based on OWL-DL, and we can formally verify and guarantee consistency using ontology reasoning tools. DL is also powerful for causal reasoning. In this study, we used the OWL transitivity axiom. With respect to chronic cellular senescence associated with type 2 diabetes mellitus, the multiple paths leading to insulin resistance were derived from the theoretical causal relationships across multiple articles. The insulin pathways have also been reported to be relevant to longevity^13^. We are planning to further investigate the effects of insulin on metabolism, the endocrine system, starvation, energy production, and growth and how these mechanisms are conserved across species. Many of the genetically homologous components of this network have been experimentally demonstrated to affect lifespan in *C. elegans*, *Drosophila*, and mice. However, the homologs of IIS/TOR components have different names in different species: for example, in *C. elegans*, the insulin-like receptor (INR) is called DAF-2, PI3K is called AGE-1, and FOXO is called DAF-16; in humans, the FOXO homolog associated with longevity is called FOXO3A^48^. Our computational unified representation could facilitate identification of common pathways and distinction of species-specific processes and causal relationships.

Aging research is becoming increasingly prominent since the risk of disease severity may increase with age. Ontological reasoning will contribute to the elucidation of the crosstalk between disease and chronic cellular senescence. As a use case for COVID-19, the present study inferred that IL-6 and IL-8 are inflammatory substances in COVID-19 and are SASP factors in cellular senescence (Fig. 4c). Thus, molecules with multiple roles in the body may be factors affecting disease severity in advanced age. In support of this, a recent review reported that SARS-CoV-2 induces hyperinflammation in SASP^49^. It was proposed that a propensity for senescence-governed immune escalation, which can be related to aging or chronic diseases, can also be acutely triggered by SARS-CoV-2 infection and lead to severe disease. Hence, the results of this study inform the commonalities between COVID-19 and cellular senescence. Here, for the processes that constitute the cellular senescence course and the COVID-19 infectious course, the common processes in both can be computationally obtained by referring to the upper classes of terms as generalized processes using the hierarchical tree of the HOIP ontology. As shown in Table S2, several common processes are related to innate immunity and inflammatory responses.

Furthermore, a generic causal network can be generated across multiple courses using generalized processes. For example, in the chronic cellular senescence course, SASP secretion and its subprocess, IL-8 signaling, might lead to chronic inflammation. In contrast, in the COVID-19 course, thrombus formation can occur through multiple pathways, from IL-8 signaling to neutrophil extracellular trap formation and NETosis. A generalized causal network of IL-8 signaling as the subprocess of SASP secretion → neutrophil extracellular trap formation/ NETosis → thrombus formation can be derived. Furthermore, IL-8 signaling can result from NFKB signaling via cGAS-STING signaling in chronic cellular senescence owing to increasing extrachromosomal telomere repeat DNA from telomere shortening or the release of CCFs due to the loss of nuclear lamina. Therefore, even if the virus is no longer in the body, thrombus formation may still occur if cellular senescence-derived IL-8 signaling occurs (Table S3). A review reported that the most destructive phase of immune activation often occurs when viral mRNA is no longer detectable^49^.

In the present study, we took diabetes as an example for other diseases and visualized metformin negatively regulating NFKB. Accordingly, for NFKB-mediated crosstalk between type 2 diabetes, chronic aging, and COVID-19, our HOIP ontology can assist the interpretation that early negative regulation of NFKB by metformin may prevent cytokine and chemokine release and chronic inflammation downstream of innate immune signaling, including the cGAS-STING pathway, and consequently suppress long COVID. Concerning metformin and COVID-19 prevention, metformin is associated with reduced COVID-19 severity in individuals with prediabetes^50^.

HOIP can also assist in preventing chronic aging via ontological reasoning as well as regulating disease factors. For example, in IL-8 signaling, which is responsible for chronic inflammation in chronic cellular senescence, ontological inference can reveal that siltuximab, which is a negative regulator of IL-8 signaling in COVID-19 drug treatment, might also be involved in the regulation of cellular senescence (Fig. S4).

By changing the conceptual level of the causal network between individual courses (mechanisms), our HOIP ontology can provide support for mechanistic interpretation by presenting potential paths across multiple mechanisms that domain experts would not have previously grasped. In other words, HOIP can be used as a reasoning technique for problem-solving drawing upon the knowledge base and thus can also provide interpretation support for experts.

In biological phenomena, causes and effects do not show one-to-one relationships. HOIP contributes to detangling the complexity of cellular senescence in diseases to make explicit the context in a computer-understandable form. By computing common processes that occur in multiple diseases and integrating all paths across diseases and cellular senescence, it might be possible to complement knowledge between mechanisms across diseases and find unknown paths for pre-emptive medicine on the computer.

Gene Ontology Causal Activity Modeling (GO-CAM)^51^ provides a structured framework that integrates multiple GO annotations to model biological systems comprehensively. GO-CAM model focuses primarily on molecular activities and their specific interactions within a target entity. The approach often emphasizes molecular-level interactions, similar to other molecular-centric pathways and databases, such as Kyoto Encyclopedia of Genes and Genomes (KEGG)^52^. However, while molecular interactions are fundamental to understanding biological processes, focusing exclusively on this level of detail can sometimes obscure the broader implications at the cellular or organismal levels. Our model, in contrast, aims to bridge this gap by exploring how molecular-level processes can lead to cellular senescence, and subsequently, how cellular senescence can manifest as clinical symptoms or diseases. This requires a cross-granularities approach to modelling causal networks that span from molecular triggers to cellular outcomes and beyond. To this end, we found it necessary to define new relationships such as ‘has result’, ‘realizes disease’, and ‘manifests symptom’. These were introduced to capture broader biological concepts and mechanisms that are not strictly molecular but also involve cellular processes and pathological manifestations. Our approach seeks to support a more holistic interpretation of cellular senescence mechanisms, encompassing and connecting multiple biological scales. Thus, while we appreciate the robust molecular foundation provided by frameworks such as GO-CAM, our ontology extends these principles to encapsulate and elucidate the complex cascade of events leading from molecular alterations to clinical outcomes. This extension is designed to provide a comprehensive understanding of the aging process, from the molecular level to its implications for aging and disease.

We created visualization maps of the ontology to support our understanding of the mechanisms of cellular senescence. Each map provides an overview of the cellular senescence course and follows the upstream causes or downstream results from a focused process of interest. For example, during cellular senescence, chromatin fragments are observed in the cytoplasm. Using the visualization, the user can trace the leakage of chromatin fragments into the cytoplasm and identify that one possible cause is a nuclear lamina defect^5^. Another possible cause is degradation of LMNB1 in lysosomes^33^. In contrast, from downstream results, the user can see the occurrence of signaling involved in the innate immune system, such as cGAS-STING signaling (Fig. 5a), which causes chronic inflammation. Our visualization of causal networks allows users to systematically understand mechanisms with varying degrees of granularity. Cytoscape provides several helpful APIs that can visualize gene interactions, such as GeneMANIA^53^. Further gene analyses are required for HOIP to use these tools and are expected to be beneficial. For example, using causal graphs based on HOIP, it is possible to determine the processes and roles of the molecules involved and trace their upstream and downstream processes. Furthermore, Cytoscape can also provide pathway data such as WikiPathways^54^; therefore, we plan to investigate the compatibility of our process-oriented description model with these forms of molecular interaction pathway data.

In conclusion, we constructed an ontology to organize knowledge and developed a representation framework model to understand cellular senescence mechanisms based on homeostasis imbalances. We demonstrated causal inferences and discussed their potential to contribute to the discovery of unknown mechanisms. We created a visualization with Cytoscape to support users in interpreting mechanisms of cellular senescence.

Limitations of the ontology include having only a few descriptions of organismal senescence, and we are only now beginning to describe symptoms specific to older adults. We will cover more chronic diseases and geriatric syndromes. Further, the ontology is currently constructed using time-consuming and labour-intensive manual annotation; semi-automating the process would improve efficiency.

The present work has some limitations. As biological networks are vast, reflecting all biological interactions and encapsulating everything within a single knowledge base are significant challenges. The approach of the present study was to extract and represent notable causal relationships from authoritative sources such as textbooks and review articles. Although we acknowledge that some sources may appear outdated, they provide valuable insights that remain relevant to understanding the concept of cellular senescence. The method aimed to provide a foundational understanding of key processes and interactions within the scope of our research. We highlight specific molecules such as PTEN and IL-6, not in isolation, but as a part of a broader attempt to map out significant pathways and interactions. For instance, the DDR pathway involves multiple processes leading from the cause (e.g., DDR) to various effects, including pathways involving p53. Our models reflect the multifaceted nature of the relationships, acknowledging that causes and effects in biology rarely have one-to-one relationships. The reason PTEN and IL-6 may appear fragmentary in Figures is the limited descriptions available in textbooks and reviews. We recognize the need to update our ontology continuously with new information. Our approach was first to establish an ontological framework that enables the computational unified representation of the mechanisms in a coherent structure. Consequently, certain aspects, such as mitochondrial functions and metabolites, have not been covered extensively. We plan to address such and other areas in subsequent versions as our ontology evolves and expands with ongoing research and feedback from the research community.

A recent article^55^ using LLM indicates low accuracy in gene annotation related to cellular senescence using KEGG. In future, we will expand the knowledge space and establish a robust framework for integrating methodologies, such as knowledge graphs and LLM-based approaches by enriching the annotation of diverse data, including genes, processes, drugs, diseases, bioresources, cells, and phenotypes. The initiative could facilitate the understanding of cellular senescence regulation and the discovery of new mechanisms. As a knowledge-sharing medium, it is anticipated that ontology will enable humans and computers to utilize knowledge from other domains, thereby benefiting multiple domains.

## Methods

### Manual annotation

We manually annotated textbooks from medicine^6,56^, pathology^7^, and biology^1,47^ relating to cellular senescence and aging mechanisms. We also curated reviews and original articles in PubMed using the Medical Subject Headings term “cellular senescence” and extracted the description from the abstract, original text, or figure legends.

### Creation and editing of the ontology

We used Protege 5.5.0^57^ as the ontology editing tool. We edited and created terms based on the HOIP. In the upper layer of the HOIP, we refer to general terms from BFO^11^ as an upper ontology. In the middle layer, biomedical terms were manually imported from external ontologies, including GO^8^, NCBI Taxonomy^22^, UBERON^16^, CL^17^, PRO^20^, ChEBI^21^, PATO^14^, HPO^15^, DOID^9^, Symptom Ontology^18^, and relational ontology (RO)^23^ (http://purl.obolibrary.org/obo/ro.owl). In the lower layer, we defined cellular senescence processes. Each entity uses owl:class and owl:SubClassOf for the is-a hierarchy. The relationships between these entities and processes are represented using the object properties (owl:ObjectProperty), defining the domain (rdfs:domain) and range (rdfs:range). We also described other molecules, anatomical entities, and cellular components relevant to cellular senescence and type 2 diabetes symptoms. Furthermore, as a logical characteristic of properties, we used a transitive property, owl:TransitiveProperty, for the causal relation (has a result). We also used the inverse relation (owl:inverseOf) for causal relationships (has cause/has result) and whole part relationships (has-part/part-of).

### Reasoning

We used Hermit^30^ and ELK^31^ as ontology reasoning tools in Protégé and executed DL queries for the HOIP. We also used SPARQL queries to check processes and relationships with other entities, including molecules. The results are presented in Supplementary Information 4.

### Visualization using Cytoscape

We used the graphical tool Cytoscape 3.9.1 for visualization. First, we built the SPARQL endpoint using Apache Fuseki (http://jena.apache.org/documentation/fuseki2/), and cellular senescence data from HOIP were obtained using SPARQL queries. Next, from the data obtained, we created tables, customized the style for nodes and edges, and transformed them into networks using CyREST as the Cytoscape API. We also exported the visualization data to NDEx from Cytoscape and published it on the web.

### Supplementary information

Supplementary Information

## Data Availability

HoIP ontology-based knowledge exporter Leaves site (https://leaves.riken.jp/)^27^: Leaves provides visualization of a course consisting of nodes representing processes and edges representing their causal relationships. Clicking on a process node in the course displays ontology information in the lower window, including article information and other related entities in each process. The visualization data are available on the NDEx website (https://www.ndexbio.org/): Cellular senescence course - Homeostasis imbalance process ontology (HOIP) (10.18119/N9T89D)^40^ Embryonic cellular senescence - Homeostasis imbalance process ontology (HOIP) (10.18119/N9PK7H)^41^ Adult cellular senescence course - Homeostasis imbalance process ontology (HOIP) (10.18119/N9Z328)^42^ Acute cellular senescence course - Homeostasis imbalance process ontology (10.18119/N96K75)^43^ Chronic cellular senescence course - Homeostasis imbalance process ontology (10.18119/N9KS4H)^44^ Chronic cellular senescence course with type 2 diabetes mellitus- Homeostasis imbalance process ontology (HOIP) (10.18119/N9G31J)^45^ Senolytics course - Homeostasis imbalance process ontology (HOIP) (10.18119/N92S4V)^46^

## Code availability

The ontology code of HOIP is available at the NCBO BioPortal ontology repository site (https://bioportal.bioontology.org/ontologies/HOIP). The license code was under Creative Commons 4.0. HOIP is also available on the GitHub website at https://github.com/yuki-yamagata/hoip.
