# Supplementary material for: Prototyping an Ontological Framework for Cellular Senescence Mechanisms: A Homeostasis Imbalance Perspective
Source: Sci Data. 2024 May 10;11:485. doi: 10.1038/s41597-024-03331-y (PMC11087592; doi:10.1038/s41597-024-03331-y)
Supplement: Supplementary file 1 — Supplementary Information [file 41597_2024_3331_MOESM1_ESM.pdf]

Title

Ontology for Cellular Senescence Mechanisms

Authors

Yuki Yamagata[1,2], Tsubasa Fukuyama[3], a, and Shuichi Onami[1,2] and Hiroshi Masuya[2,4]

Affiliations

1. Laboratory for Developmental Dynamics, RIKEN Center for Biosystems Dynamics Research, 2-2-3 Minatojima-minamimachi, Chuo-ku, Kobe, Hyogo 650-0047, Japan
2. Life Science Data Sharing Unit, Infrastructure Research and Development Division, RIKEN Information R&D and Strategy Headquarters, 2-2-3 Minatojima-minamimachi, Chuo-ku, Kobe, Hyogo 650-0047, Japan
3. AXIOHELIX CO., LTD., 8F Kubota Bldg., 1-12-17 Kandaizumicho, Chiyoda-ku, Tokyo, 101-0024 Japan
4. Integrated Bioresource Information Division, RIKEN BioResource Research Center, Kouyadai 3-1-1 Tsukuba, Ibaraki 305-0074 Japan

corresponding authors: Yuki Yamagata (yuki.yamagata@riken.jp), Hiroshi Masuya (hiroshi.masuya@riken.jp)

Supplementary Table S1

Main cellular senescence-related terms defined in the HoIP ontology.

Supplementary Table S2

Common processes in chronic cellular senescence course (HOIP:0060195) and COVID-19 infectious courses

Supplementary Table S3

General causal network across the chronic cellular senescence course (HOIP:0060195) and COVID-19 infectious courses

Supplementary Figure S1

A unified representation of cellular senescence courses.

Supplementary Figure S2
Examples of validation of causal networks including part of the whole (‘has part’) relations of processes by ontology reasoning tool ELK in Protégé.

Supplementary Figure S3
Visualization workflow of cellular senescence courses from the HoIP ontology.

Supplementary Figure S4
Causal inference of cause of possible negative regulation of IL-8 signaling by the ontology reasoning tool ELK in Protégé.

Supplementary Information 1
Logical equation of senescence-associated secretory phenotype (SASP) secretion in chronic cellular senescence course

Supplementary Information 2
Logical equation of chronic cellular senescence course

Supplementary Information 3
SPARQL query to find processes that compose chronic cellular senescence course

Supplementary Information 4
SPARQL query to find other relationships between processes and with other entities, such as molecules in various cellular senescence courses.

Supplementary Table S1

Main cellular senescence-related terms defined in the HoIP ontology.

HOIP: http://purl.bioontology.org/ontology/HOIP

E.g., HOIP:0060195 http://purl.bioontology.org/ontology/HOIP/HOIP_0060195

PR: http://purl.obolibrary.org/obo/pr.owl

E.g., PR:P42772 http://purl.obolibrary.org/obo/PR_P42772

Process URI	Process	Relation	Molecule URI	Molecule
HOIP:0060269	TGF beta signaling [embryonic cellular senescence]	has agent	PR:000000046	TGF-beta
HOIP:0060280	p15 signaling (transient) [embryonic cellular senescence]	has agent	PR:P42772	hCDKN1B
HOIP:0060290	SMAD signaling [embryonic cellular senescence]	has agent	PR:000000027	smad protein
HOIP:0060299	cellular senescence (transient) [embryonic cellular senescence course]			
HOIP:0060399	embryonic pattern specification [embryonic cellular senescence]			
HOIP:0060380	response to tumor cell proliferation [acute cellular senescence]			
HOIP:0060600	PTEN loss [acute cellular senescence]	has input	PR:P60484	hPTEN

HOIP:0060411	PI3K signaling (transient) [acute cellular senescence]	has agent	PR:000026439	phosphoinositide 3-kinase complex (human)
HOIP:0060409	AKT signaling (transient) [acute cellular senescence]	has agent	PR:P31749	hAKT1
HOIP:0060608	increasing demand for oncogenic stress response [acute cellular senescence]			
HOIP:0060338	DNA damage response signaling [acute cellular senescence]			
HOIP:0060092	p16 signaling (transient) [acute cellular senescence]	has agent	PR:000030011	hCDKN2A
HOIP:0060291	p21 signaling (transient) [acute cellular senescence]	has agent	PR:P38936	hCDKN1A
HOIP:0060241	cellular senescence (transient) [acute cellular senescence course]			
HOIP:0060498	senescence-associated secretory phenotype (SASP) secretion (transient) [acute cellular senescence]			
HOIP:0060311	tissue remodeling [acute cellular senescence]			
HOIP:0060298	tissue repair [acute cellular senescence]			

HOIP:0060573	positive regulation of tumor cell apoptotic process [acute cellular senescence]			
HOIP:0060230	negative regulation of tumor cell proliferation [acute cellular senescence]			
HOIP:0060091	mitochondrial dysfunction [chronic cellular senescence]			
HOIP:0060050	telomere shortening [chronic cellular senescence]			
HOIP:0060266	reactive oxygen species biosynthetic process (sustained) [chronic cellular senescence]			
HOIP:0060162	senescence-associated heterochromatin focus formation [chronic cellular senescence]			
HOIP:0060102	senescence-associated secretory phenotype (SASP) secretion [chronic cellular senescence]			
HOIP:0060639	IL-6 signaling [chronic cellular senescence]	has agent	PR:P05231	interleukin-6 (human)
HOIP:0060640	CXCL8 signaling [chronic cellular senescence]	has agent	PR:P10145	interleukin-8 (human)
HOIP:0060113	chronic inflammation [chronic cellular senescence]			

HOIP:0060174	positive regulation of carcinogenesis [chronic cellular senescence]			

Supplementary Table S2

Common processes in chronic cellular senescence course (HOIP:0060195) and COVID-19 infectious courses:

- COVID-19 infectious course (HOIP:0037002)
- severe COVID-19 infectious course (HOIP:0039222)
- COVID-19 course associated with acute respiratory distress syndrome (HOIP:0036006)

Process URI	Process
HOIP:0000102	increasing demand for response to oxidative stress
HOIP:0000192	inflammatory response
HOIP:0000219	fibrosis
HOIP:0000540	inflammatory cell infiltration
HOIP:0000761	NFKB signaling
HOIP:0002073	organ malfunction
HOIP:0002361	mitochondrial dysfunction
HOIP:0004327	IL-6 signaling
HOIP:0004451	CCL2 signaling
HOIP:0004657	IL1 signaling
HOIP:0036193	IRF3 activation
HOIP:0040446	decreasing stem cell
HOIP:0040448	negative regulation of tissue regeneration
HOIP:0041903	CXCL8 signaling
GO:0002532	production of molecular mediator involved in inflammatory response
GO:0032606	type I interferon production
GO:0043065	positive regulation of apoptotic process
GO:0045089	positive regulation of innate immune response
GO:0050729	positive regulation of inflammatory response
GO:1903409	reactive oxygen species biosynthetic process
NCIT:C16507	DNA damage

HOIP: http://purl.bioontology.org/ontology/HOIP

E.g., HOIP:0000102 http://purl.bioontology.org/ontology/HOIP/HOIP_0000102

GO: http://purl.obolibrary.org/obo/GO.owl

E.g., GO:002532 http://purl.obolibrary.org/obo/GO_0002532

NCIT: http://purl.obolibrary.org/obo/NCIT.owl

E.g., NCIT: C_16507 http://purl.obolibrary.org/obo/NCIT_C_16507

Supplementary Table S3

General causal network across the chronic cellular senescence course (HOIP:0060195) and COVID-19 infectious courses:

- COVID-19 infectious course (HOIP:0037002)
- severe COVID-19 infectious course (HOIP:0039222)
- COVID-19 course associated with acute respiratory distress syndrome (HOIP:0036006)

Process (cause)	Relation	Process (result)
telomere shortening	has result	increasing extrachromosomal telomeric repeat DNA
increasing extrachromosomal telomeric repeat DNA	has result	cGAS signaling
loss of nuclear lamina	has result	release of cytosolic chromatin fragments (CCFs)
release of cytosolic chromatin fragments (CCFs)	has result	cGAS activation
cGAS activation	has result	cGAS signaling
cGAS signaling	has result	STING signaling
STING signaling	has result	NFKB signaling
NFKB signaling	has result	CXCL8 signaling
NFKB signaling	has result	senescence-associated secretory phenotype (SASP) secretion
senescence-associated secretory phenotype (SASP) secretion	has part	CXCL8 signaling
senescence-associated secretory phenotype (SASP) secretion	has result	chronic inflammation
CXCL8 signaling	has result	chronic inflammation
CXCL8 signaling	has result	neutrophil chemotaxis
neutrophil chemotaxis	has result	neutrophil activation involved in inflammatory response
neutrophil activation involved in inflammatory response	has result	neutrophil extracellular trap formation
neutrophil extracellular trap formation	has result	Netosis

Netosis	has result	positive regulation of blood coagulation
positive regulation of blood coagulation	has result	blood coagulation
blood coagulation	has result	positive regulation of thrombus formation
positive regulation of thrombus formation	has result	thrombus formation

Supplementary Figure S1

A unified representation of cellular senescence courses.

(a)

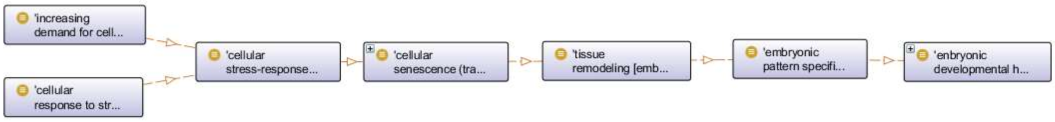

"'cellular response to stress (transient) [embryonic cellular senescence]'"
-> "'cellular stress-response imbalance [embryonic cellular senescence]'" [label="'has result'(Subclass some)"]
"'increasing demand for cellular stress response [embryonic cellular senescence]'"
-> "'cellular stress-response imbalance [embryonic cellular senescence]'" [label="'has result'(Subclass some)"]
"'cellular stress-response imbalance [embryonic cellular senescence]'"
-> **"'cellular senescence (transient)** [embryonic cellular senescence course]'" [label="'has result'(Subclass some)"]
"'cellular senescence (transient) [embryonic cellular senescence course]'"
-> "'tissue remodeling [embryonic cellular senescence]'" [label="'has result'(Subclass some)"]
"'tissue remodeling [embryonic cellular senescence]'" -> "'embryonic pattern specification [embryonic cellular senescence]'" [label="'has result'(Subclass some)"]
"'embryonic pattern specification [embryonic cellular senescence]'" -> "'enbryonic developmental homeorhesis process [embryonic cellular senescence]'" [label="'has result'(Subclass some)"]

(a) The upper figure shows a screenshot of OntoGraf in Protégé displaying the imbalance model in the embryonic cellular senescence course, with 'Arc Types' selected to be causal relationships ("has result") for the cellular senescence process. Lower indicates a partial excerpt of the causal relationships (shown in "->") using the 'export DOT' function from OntoGraf. In embryonic cellular senescence, the cellular-stress imbalance could result in cellular senescence (transient). Consequently, tissue remodeling could cause pattern formation specification.

(b)

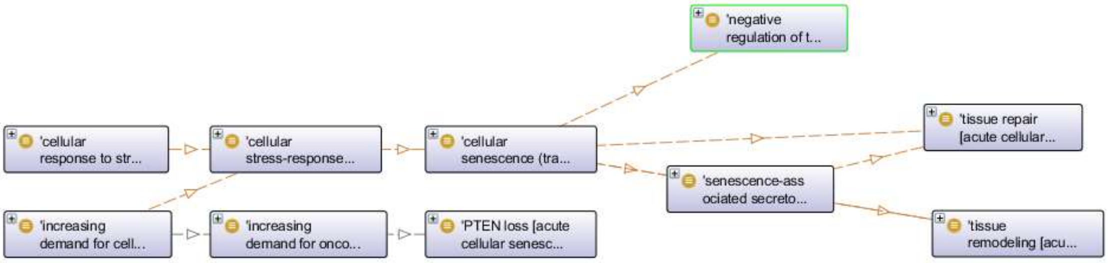

'''increasing demand for cellular stress response (transient) [acute cellular senescence]''' -> '''increasing demand for oncogenic stress response [acute cellular senescence]''' [label='''has part'(Subclass some)'']
'''increasing demand for oncogenic stress response [acute cellular senescence]''' -> **'''PTEN loss** [acute cellular senescence]''' [label='''has part'(Subclass some)'']
'''increasing demand for cellular stress response (transient) [acute cellular senescence]''' -> '''cellular stress-response imbalance [acute cellular senescence]''' [label='''has result'(Subclass some)'']
'''cellular stress-response imbalance [acute cellular senescence]''' -> **'''cellular senescence (transient)** [acute cellular senescence course]''' [label='''has result'(Subclass some)'']
'''cellular senescence (transient) [acute cellular senescence course]''' -> '''tissue remodeling [acute cellular senescence]''' [label='''has result'(Subclass some)'']
'''cellular response to stress (transient) [acute cellular senescence]''' -> '''cellular stress-response imbalance [acute cellular senescence]''' [label='''has result'(Subclass some)'']
'''cellular senescence (transient) [acute cellular senescence course]''' -> '''tissue repair [acute cellular senescence]''' [label='''has result'(Subclass some)'']
'''cellular senescence (transient) [acute cellular senescence course]''' -> '''senescence-associated secretory phenotype (SASP) secretion (transient) [acute cellular senescence]''' [label='''has result'(Subclass some)'']
'''senescence-associated secretory phenotype (SASP) secretion (transient) [acute cellular senescence]''' -> '''tissue remodeling [acute cellular senescence]''' [label='''has result'(Subclass some)'']
'''senescence-associated secretory phenotype (SASP) secretion (transient) [acute cellular senescence]''' -> **'''tissue repair** [acute cellular senescence]''' [label='''has result'(Subclass some)'']
'''cellular senescence (transient) [acute cellular senescence course]''' -> **'''negative regulation of tumor cell proliferation** [acute cellular senescence]''' [label='''has result'(Subclass some)'']

(b) The upper figure depicts causal relationships introduced by the imbalance model in the acute cellular senescence course using OntoGraf in Protégé. In the case of acute cellular senescence, there is increasing demand for the oncogenic stress response, which has the subprocess PTEN loss, and the imbalance could lead to cellular senescence (transient), tissue repair, and negative regulation of tumor proliferation.

(c)

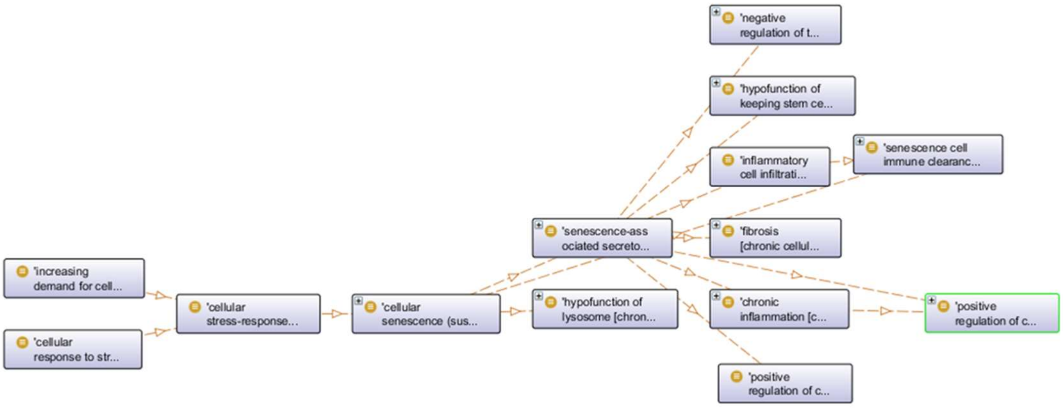

"'increasing demand for cellular stress response (sustained) [chronic cellular senescence]'"
-> "'cellular stress-response imbalance [chronic cellular senescence]'" [label="'has result'(Subclass some)"]
"'cellular stress-response imbalance [chronic cellular senescence]'"
-> **"'cellular senescence (sustained)** [chronic cellular senescence course]'" [label="'has result'(Subclass some)"]
"'cellular senescence (sustained) [chronic cellular senescence course]'"
-> "'senescence cell immune clearance (low) [chronic cellular senescence]'" [label="'has result'(Subclass some)"]
"'inflammatory cell infiltration [chronic cellular senescence]'"
-> "'senescence cell immune clearance (low) [chronic cellular senescence]'" [label="'has result'(Subclass some)"]
"'cellular senescence (sustained) [chronic cellular senescence course]'"
-> "'senescence-associated secretory phenotype (SASP) secretion [chronic cellular senescence]'" [label="'has result'(Subclass some)"]
"'senescence-associated secretory phenotype (SASP) secretion [chronic cellular senescence]'"
-> "'negative regulation of tissue regeneration [chronic cellular senescence]'" [label="'has result'(Subclass some)"]
"'senescence-associated secretory phenotype (SASP) secretion [chronic cellular senescence]'"
-> "'positive regulation of cellular senescence in neighboring cell [chronic cellular senescence]'" [label="'has result'(Subclass some)"]
"'cellular senescence (sustained) [chronic cellular senescence course]'"
-> "'hypofunction of lysosome [chronic cellular senescence]'" [label="'has result'(Subclass some)"]
"'senescence-associated secretory phenotype (SASP) secretion [chronic cellular senescence]'" -> "'positive regulation of carcinogenesis [chronic cellular senescence]'" [label="'has result'(Subclass some)"]
"'senescence-associated secretory phenotype (SASP) secretion [chronic cellular senescence]'"
-> **"'chronic inflammation** [chronic cellular senescence]'" [label="'has result'(Subclass some)"]
"'chronic inflammation [chronic cellular senescence]'"
-> "'positive regulation of carcinogenesis [chronic cellular senescence]'" [label="'has result'(Subclass some)"]

(c) The upper figure shows causal relationships introduced by the imbalance model in the chronic cellular senescence course using OntoGraf in Protégé. In chronic cellular senescence, the imbalance could cause persistent cellular senescence, which might lead to SASP secretion, which could cause chronic inflammation.

Supplementary Figure S2

Examples of validation of causal networks including part of the whole ('has part') relations of processes by ontology reasoning tool ELK in Protégé.

In reasoning, direct and indirect causal relationships are inferred using the transitive property (owl: Transitive Property).

(a)

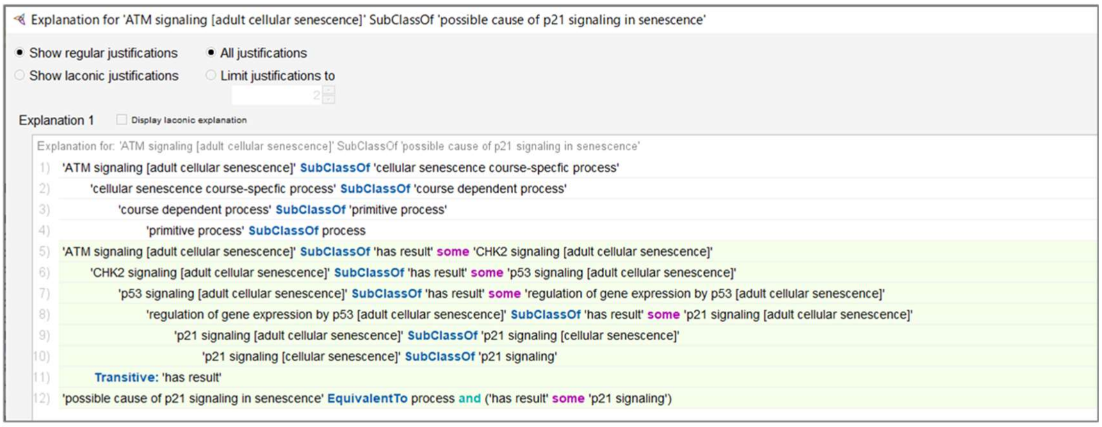

(b)

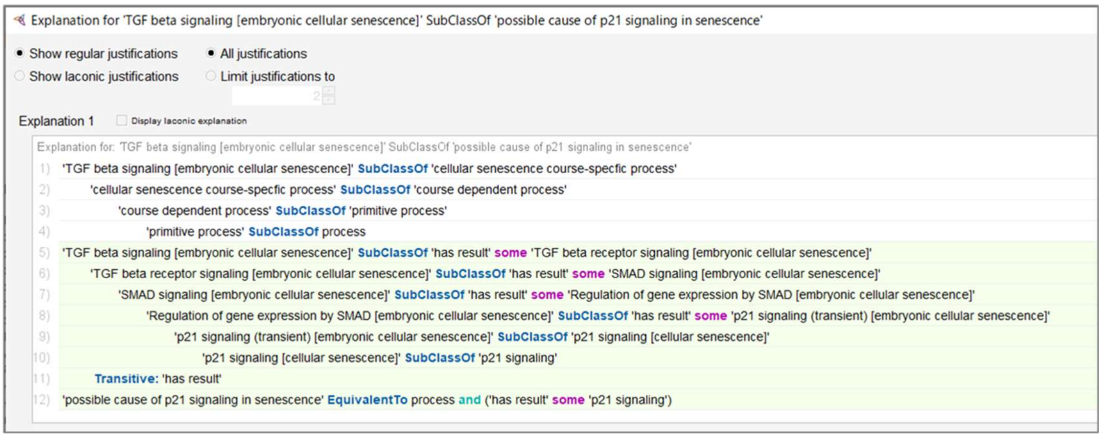

(a) By inference, a path from ATM signaling (HOIP:0060610) to p21 signaling (HOIP:0060325) could occur in adult cellular senescence. One of the results inferred that ATM signaling (HOIP:0060110) could result in CHK2 signaling (HOIP:0060145), p53 signaling (HOIP:0060055), regulation of gene expression by p53 (HOIP:0060065), and p21 signaling (HOIP:0060325).

(b) Inference of p21 signaling (HOIP:0060548) in embryonic cellular senescence (HOIP:0060267). The result indicated that TGF beta signaling (HOIP:0060269), TGF beta receptor signaling (HOIP:0060270), and SMAD signaling (HOIP:0060290) could lead to the regulation of gene expression by SMAD (HOIP:0060276), which might result in p21 signaling (HOIP:0060548).

(c)

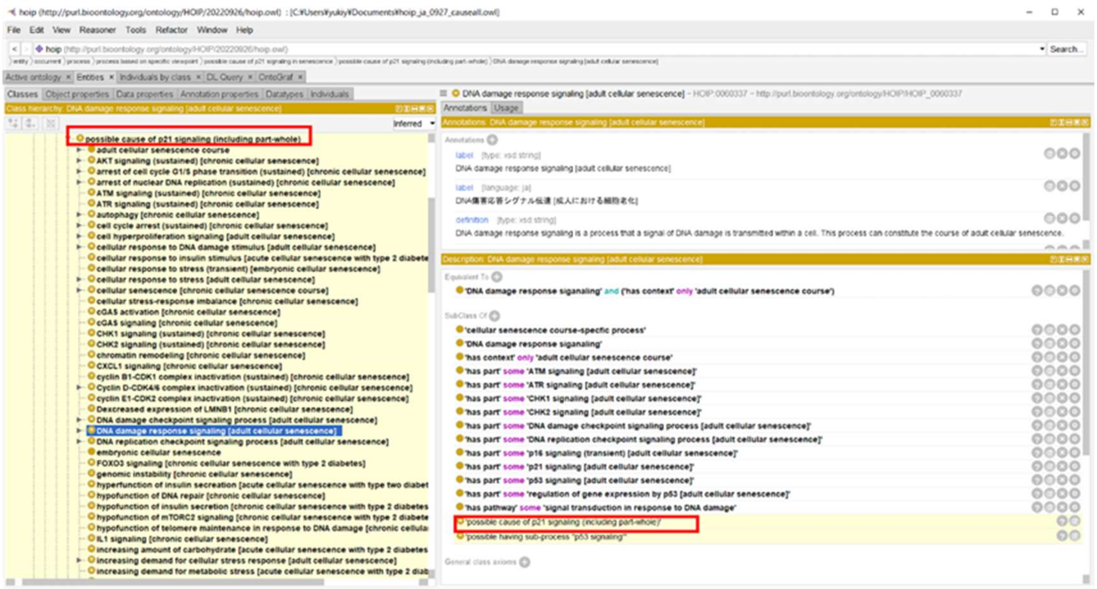

(c) A screenshot for a list of the results of causal inference of the ontology reasoner ELK in Protégé software, including part of whole relations of processes and p21 signaling (HOIP:0060325) in the adult cellular senescence course used in the ELK reasoner in Protégé.
The upper red frame shows the possible cause of p21 signaling [adult cellular senescence] for validating causal networks. Inferred information is shown on a yellow background (lower red frame). The DNA damaging response signaling (HOIP:0060337) is inferred to be a subclass of possible cause of p21 signaling [adult cellular senescence]

(d)

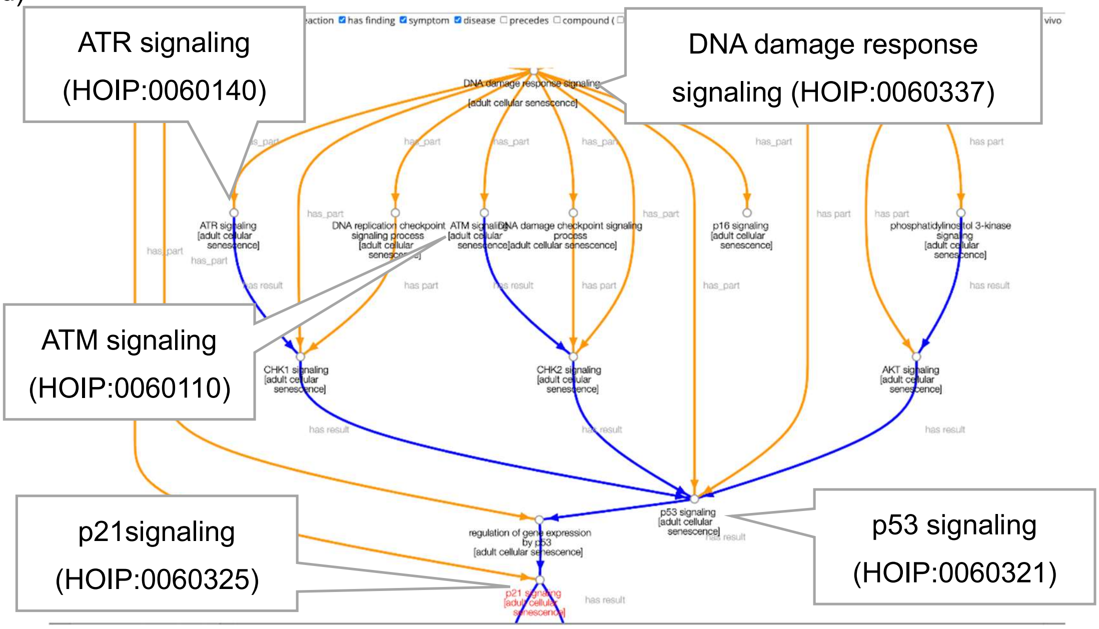

(d) Representation of the inference results in Leaves (https://leaves.riken.jp/). Each node shows the process, and each edge shows the relationship (orange: has part, blue:/has result) between processes.

(e)

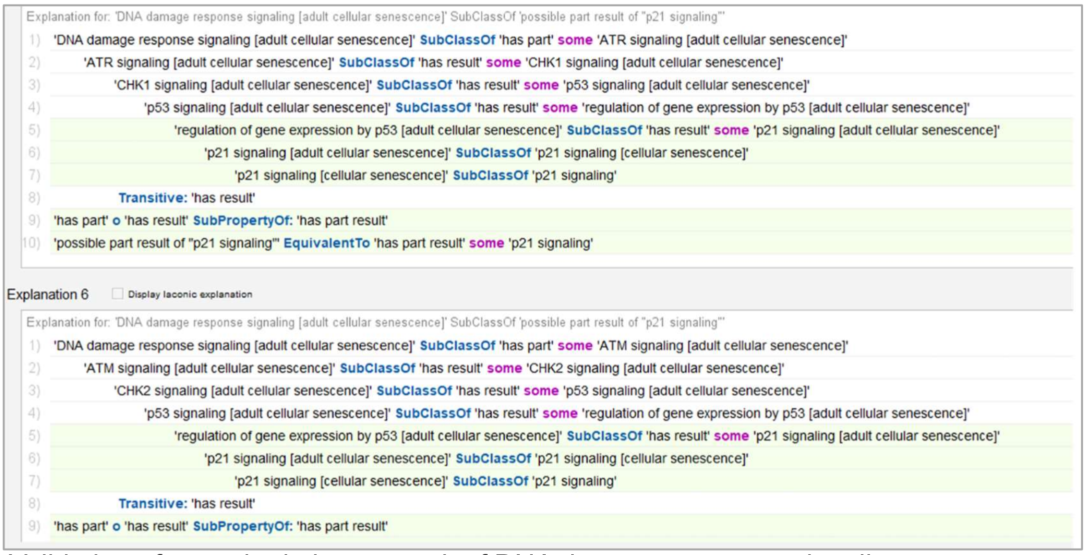

Explanation for: 'DNA damage response signaling [adult cellular senescence]' SubClassOf 'possible part result of "p21 signaling"'

1) 'DNA damage response signaling [adult cellular senescence]' **SubClassOf** 'has part' **some** 'ATR signaling [adult cellular senescence]'
2) 'ATR signaling [adult cellular senescence]' **SubClassOf** 'has result' **some** 'CHK1 signaling [adult cellular senescence]'
3) 'CHK1 signaling [adult cellular senescence]' **SubClassOf** 'has result' **some** 'p53 signaling [adult cellular senescence]'
4) 'p53 signaling [adult cellular senescence]' **SubClassOf** 'has result' **some** 'regulation of gene expression by p53 [adult cellular senescence]'
5) 'regulation of gene expression by p53 [adult cellular senescence]' **SubClassOf** 'has result' **some** 'p21 signaling [adult cellular senescence]'
6) 'p21 signaling [adult cellular senescence]' **SubClassOf** 'p21 signaling [cellular senescence]'
7) 'p21 signaling [cellular senescence]' **SubClassOf** 'p21 signaling'
8) **Transitive:** 'has result'
9) 'has part' **o** 'has result' **SubPropertyOf:** 'has part result'
10) 'possible part result of "p21 signaling"' **EquivalentTo** 'has part result' **some** 'p21 signaling'

Explanation 6 ☐ Display laconic explanation

Explanation for: 'DNA damage response signaling [adult cellular senescence]' SubClassOf 'possible part result of "p21 signaling"'

1) 'DNA damage response signaling [adult cellular senescence]' **SubClassOf** 'has part' **some** 'ATM signaling [adult cellular senescence]'
2) 'ATM signaling [adult cellular senescence]' **SubClassOf** 'has result' **some** 'CHK2 signaling [adult cellular senescence]'
3) 'CHK2 signaling [adult cellular senescence]' **SubClassOf** 'has result' **some** 'p53 signaling [adult cellular senescence]'
4) 'p53 signaling [adult cellular senescence]' **SubClassOf** 'has result' **some** 'regulation of gene expression by p53 [adult cellular senescence]'
5) 'regulation of gene expression by p53 [adult cellular senescence]' **SubClassOf** 'has result' **some** 'p21 signaling [adult cellular senescence]'
6) 'p21 signaling [adult cellular senescence]' **SubClassOf** 'p21 signaling [cellular senescence]'
7) 'p21 signaling [cellular senescence]' **SubClassOf** 'p21 signaling'
8) **Transitive:** 'has result'
9) 'has part' **o** 'has result' **SubPropertyOf:** 'has part result'

(e) Validation of causal relation network of DNA damage response signaling (HOIP:0060337) by the ontology reasoning tool ELK in Protégé. The results inferred that DNA damage response signaling (HOIP:0060337) has subprocesses, ATR signaling (HOIP:0060140) (upper figure) or ATM signaling (HOIP:0060110) (lower figure), which could cause CHK1 signaling (HOIP:0060144) (upper figure)/CHK2 signaling (HOIP:0060145) (lower figure). This could then result in p53 signaling (HOIP:0060055), regulation of gene expression by p53 (HOIP:0060065), and p21 signaling (HOIP:0060325).

Supplementary Figure S3

Visualization workflow of cellular senescence courses from HoIP ontology.

- The ontology data are stored in an RDF triple store using Apache Jena Fuseki to construct the SPARQL endpoint.
- Necessary information for visualization is dynamically acquired via SPARQL queries
- Concerning data processing, information tables are made by Python-based Jupiter Notebook
- Graphs are generated semi-automatically from the table by Cytoscape
- Uploading data to NDEx and publishing the data on the web.

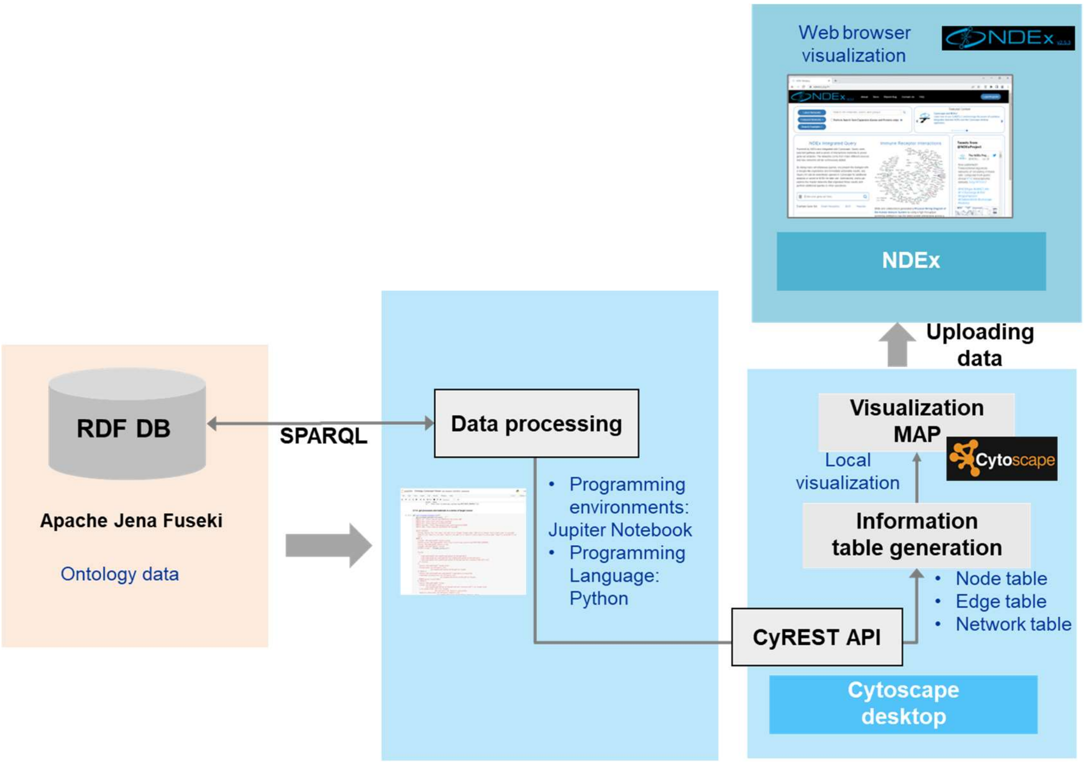

Supplementary Figure S4

Causal inference of cause of possible negative regulation of CXCL8/IL-8 signaling by the ontology reasoning tool ELK in Protégé.

Inference results in DL query.

(a) Inference shows the query for finding negative regulation of IL-8 signaling.

(b) The result shows siltuximab as the agent of negative regulation of IL-8 signaling.

(a)

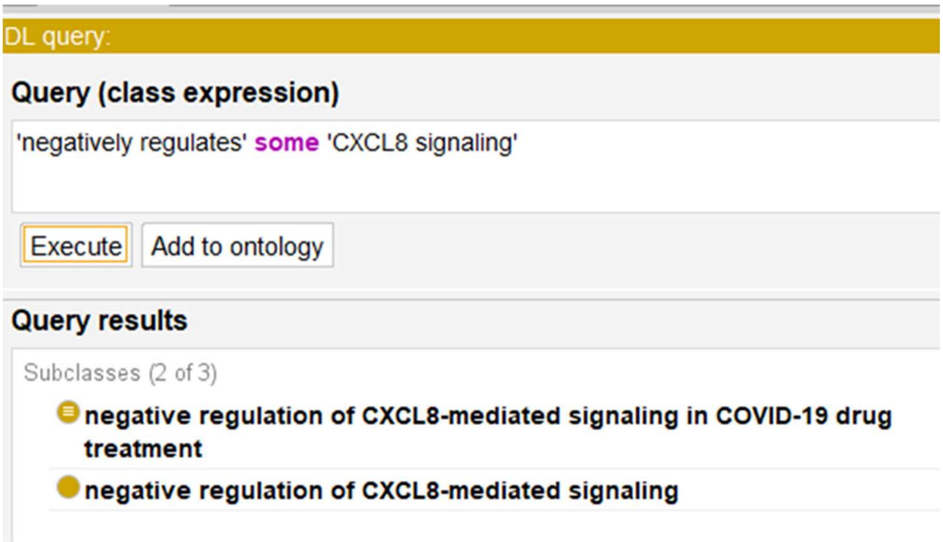

(b)

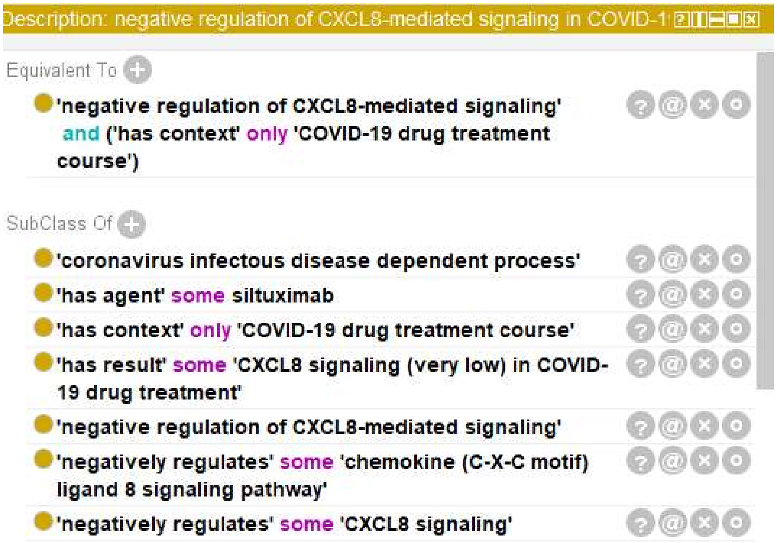

Supplementary Information 1

Logical equation of senescence-associated secretory phenotype (SASP) secretion in chronic cellular senescence course

HoIP ontology is described in the OWL DL (Web Ontology Language - Description Logic) format, leveraging its compatibility with description logic principles. We utilized the Description Logic (DL) syntax, employing constructors such as subsumption (⊆), intersection (∩), universal restriction (∀), and existential restriction (∃).

The process senescence-associated secretory phenotype (SASP) secretion in chronic cellular senescence (HOIP:0060102) can be expressed by the following equation (some parts are omitted):

senescence-associated secretory phenotype (SASP) secretion in chronic cellular senescence
⊆(senescence-associated secretory phenotype (SASP) secretion in adult cellular senescence
∩ ∃has part. CXCL8 signaling
∩ ∃has part. IL-6 signaling
∩ ∃has output. interleukin-6 (human) ∩ (∃has role. SASP factor)
∩ ∃has output. interleukin-8 (human) ∩ (∃has role. SASP factor)
∩ ∃has result. chronic inflammation
∩ ∃has result. fibrosis
∩ ∃has result. hypofunction of keeping stem cell niche
∩ ∃has result. negative regulation of tissue regeneration
∩ ∃has result. positive regulation of carcinogenesis
∩ ∃has result. positive regulation of cellular senescence in neighboring cell
∩ ∃occurs in. senescent cell
∩ ∀has context. chronic cellular senescence)

Supplementary Information 2

Logical equation of chronic cellular senescence course

This study describes the chronic cellular senescence course (HOIP_0060195) as a subclass of the adult cellular senescence course. The processes defined in each cellular senescence course were enumerated using the 'has part' relation. For example, processes such as 'chronic inflammation (HOIP_0060113),' were the components of chronic cellular senescence. The equation is shown as follows:

Chronic cellular senescence
⊆ (adult cellular senescence course
∩ has part. accumulation of senescent cell (sustained) [chronic cellular senescence]
∩ has part. aging [chronic cellular senescence]
∩ has part. AKT signaling (sustained) [chronic cellular senescence]
∩ has part. arrest of cell cycle G1/S phase transition (sustained) [chronic cellular senescence]
∩ has part. arrest of nuclear DNA replication (sustained) [chronic cellular senescence]
∩ has part. ATM signaling (sustained) [chronic cellular senescence]
∩ has part. ATR signaling (sustained) [chronic cellular senescence]
∩ has part. autophagy [chronic cellular senescence]
∩ has part. CCL2 signaling [chronic cellular senescence]
∩ has part. cell cycle arrest (sustained) [chronic cellular senescence]
∩ has part. cell hyperproliferation signaling (sustained) [chronic cellular senescence]
∩ has part. cellular response to DNA damage stimulus (sustained) [chronic cellular senescence]
∩ has part. cellular response to stress (sustained) [chronic cellular senescence]
∩ has part. cellular senescence (sustained) [chronic cellular senescence course]
∩ has part. cellular stress-response imbalance [chronic cellular senescence]
∩ has part. cGAS activation [chronic cellular senescence]
∩ has part. cGAS signaling [chronic cellular senescence]
∩ has part. CHK1 signaling (sustained) [chronic cellular senescence]
∩ has part. CHK2 signaling (sustained) [chronic cellular senescence]
∩ has part. chromatin remodeling [chronic cellular senescence]
∩ has part. chronic inflammation [chronic cellular senescence]
∩ has part. CXCL1 signaling [chronic cellular senescence]
∩ has part. CXCL8 signaling [chronic cellular senescence]
∩ has part. cyclin B1-CDK1 complex inactivation (sustained) [chronic cellular senescence]

∩ has part. Cyclin D-CDK4/6 complex inactivation (sustained) [chronic cellular senescence]
∩ has part. cyclin E1-CDK2 complex inactivation (sustained) [chronic cellular senescence]
∩ has part. Decreased expression of LMNB1 [chronic cellular senescence]
∩ has part. decreasing PARP1 level [chronic cellular senescence]
∩ has part. decreasing stem cell [chronic cellular senescence]
∩ has part. DNA damage (sustained) [chronic cellular senescence]
∩ has part. DNA damage checkpoint signaling process [chronic cellular senescence]
∩ has part. DNA damage response siganaling [chronic cellular senescence]
∩ has part. DNA replication checkpoint signaling process [chronic cellular senescence]
∩ has part. fibrosis [chronic cellular senescence]
∩ has part. genomic instability [chronic cellular senescence]
∩ has part. HMGB1 activation [chronic cellular senescence]
∩ has part. hypofunction of DNA repair [chronic cellular senescence]
∩ has part. hypofunction of keeping stem cell niche [chronic cellular senescence]
∩ has part. hypofunction of lysosome [chronic cellular senescence]
∩ has part. hypofunction of telomere maintenance in response to DNA damage [chronic cellular senescence]
∩ has part. IL-1 signaling [chronic cellular senescence]
∩ has part. IL-6 signaling [chronic cellular senescence]
∩ has part. increasing demand for cellular stress response (sustained) [chronic cellular senescence]
∩ has part. increasing demand for oncogenic stress response [chronic cellular senescence]
∩ has part. increasing demand for response to oxidative stress [chronic cellular senescence]
∩ has part. increasing extrachromosomal telomeric repeat DNA [chronic cellular senescence]
∩ has part. increasing number of cell division [chronic cellular senescence]
∩ has part. inflammatory cell infiltration [chronic cellular senescence]
∩ has part. innate immune response [chronic cellular senescence]
∩ has part. interleukin-1 alpha production [chronic cellular senescence]
∩ has part. IRF activation [chronic cellular senescence]
∩ has part. lipofuscin deposition [chronic cellular senescence]
∩ has part. LMNB1degradation in lysosome　[chronic cellular senescence]
∩ has part. loss of nuclear lamina [chronic cellular senescence]
∩ has part. loss of telomere maintenance via telomeraseis in somatic cell [chronic cellular senescence]

∩ has part. Mitochondrial DNA damage [chronic cellular senescence]
∩ has part. mitochondrial dysfunction [chronic cellular senescence]
∩ has part. mitochondrial membrane fluidity change [chronic cellular senescence]
∩ has part. MK2 signaling [chronic cellular senescence]
∩ has part. mTOR signaling [chronic cellular senescence]
∩ has part. negative regulation of apoptotic process [chronic cellular senescence]
∩ has part. negative regulation of cell cycle G1/S phase transition [chronic cellular senescence]
∩ has part. negative regulation of cell cycle G2/M phase transition (sustained) [chronic cellular senescence]
∩ has part. negative regulation of double-strand break repair via homologous recombination [chronic cellular senescence]
∩ has part. negative regulation of double-strand break repair via nonhomologous end joining
∩ has part. negative regulation of double-strand break repair via nonhomologous end joining [chronic cellular senescence]
∩ has part. negative regulation of nuclear cell cycle DNA replication (sustained) [chronic cellular senescence]
∩ has part. negative regulation of telomere fusion [chronic cellular senescence]
∩ has part. negative regulation of tissue regeneration [chronic cellular senescence]
∩ has part. negative regulation of transcription by RNA polymerase II E2F (sustained) [chronic cellular senescence]
∩ has part. NfkB signaling [chronic cellular senescence]
∩ has part. oncogene activation [chronic cellular senescence]
∩ has part. organ malfunction [chronic cellular senescence]
∩ has part. p16 signaling (sustained) [chronic cellular senescence]
∩ has part. p21 signaling (sustained) [chronic cellular senescence]
∩ has part. p38 signaling [chronic cellular senescence]
∩ has part. p53 signaling [chronic cellular senescence]
∩ has part. paracrine signaling [chronic cellular senescence]
∩ has part. phospholipid peroxidation in mitochondrial membrane [chronic cellular senescence]
∩ has part. PI3K signaling (sustained) [chronic cellular senescence]
∩ has part. positive regulation of apoptotic process [chronic cellular senescence]
∩ has part. positive regulation of autophagy [chronic cellular senescence]
∩ has part. positive regulation of carcinogenesis [chronic cellular senescence]

∩ has part. positive regulation of cell proliferation [chronic cellular senescence]
∩ has part. positive regulation of cellular senescence in neighboring cell [chronic cellular senescence]
∩ has part. positive regulation of inflammatory response [chronic cellular senescence]
∩ has part. positive regulation of innate immune response [chronic cellular senescence]
∩ has part. positive regulation of NfkB signaling [chronic cellular senescence]
∩ has part. positive regulation of reactive oxygen species biosynthetic process [chronic cellular senescence]
∩ has part. positive regulation of senescence-associated heterochromatin focus formation [chronic cellular senescence]
∩ has part. positive regulation of senescence-associated secretory phenotype (SASP) secretion [chronic cellular senescence]
∩ has part. positive regulation of tumor cell proliferation [chronic cellular senescence]
∩ has part. protein production associated with SASP [chronic cellular senescence]
∩ has part. RB activation (sustained) [chronic cellular senescence]
∩ has part. reactive oxygen species biosynthetic process (sustained) [chronic cellular senescence]
∩ has part. regulation of cell cycle [chronic cellular senescence]
∩ has part. regulation of gene expression by p53 [chronic cellular senescence]
∩ has part. release of cytosolic chromatin fragments (CCFs) [chronic cellular senescence]
∩ has part. response to metabolic stress [chronic cellular senescence]
∩ has part. response to oxidative stress (sustained) [chronic cellular senescence]
∩ has part. response to tumor cell proliferation [chronic cellular senescence]
∩ has part. SA-β-GAL acculumation [chronic cellular senescence]
∩ has part. SASP related protein expression by NF-kappa B [chronic cellular senescence]
∩ has part. senescence cell immune clearance (low) [chronic cellular senescence]
∩ has part. senescence-associated heterochromatin focus formation [chronic cellular senescence]
∩ has part. senescence-associated secretory phenotype (SASP) secretion [chronic cellular senescence]
∩ has part. STING signaling [chronic cellular senescence]
∩ has part. telomere shortening [chronic cellular senescence]
∩ has part. tissue malfunction [chronic cellular senescence]
∩ has part. tumor cell proliferation [chronic cellular senescence]
∩ has part. type I interferon production [chronic cellular senescence])

Supplementary Information 3

SPARQL query:

```
# Find the processes that compose HOIP_0060195 chronic cellular senescence course

PREFIX rdfs: <http://www.w3.org/2000/01/rdf-schema#>
PREFIX rdf:<http://www.w3.org/1999/02/22-rdf-syntax-ns#>
PREFIX owl:<http://www.w3.org/2002/07/owl#>
PREFIX hoip:<http://purl.bioontology.org/ontology/HOIP/>
PREFIX obo:<http://purl.obolibrary.org/obo/>
SELECT DISTINCT ?course_uri ?course_label ?process_uri ?process_label
WHERE
{
   ?course_uri rdfs:label ?course_label .
   FILTER (?course_uri = hoip:HOIP_0060195)
   FILTER (lang(?course_label) = '')
   hoip:HOIP_0060195 rdfs:subClassOf [
                     a owl:Restriction ;
                     owl:onProperty obo:BFO_0000051 ;
                     owl:someValuesFrom ?process_uri
                     ] .
   ?process_uri rdfs:subClassOf+ hoip:HOIP_0060021.
   ?process_uri rdfs:label ?process_label .
   FILTER (lang(?process_label) = '')
}
```

**ID**	**Term label**
HOIP_0060195	chronic cellular senescence course
BFO_0000051	has part
HOIP_0060021	cellular senescence course-specific process

Supplementary Information 4

SPARQL query to find other relationships between processes and with other entities such as molecules in various cellular senescence courses.

1. Find the direct sub-processes of HOIP_0060337 DNA damage response signaling [adult cellular senescence]

```
PREFIX rdfs: <http://www.w3.org/2000/01/rdf-schema#>
PREFIX rdf:<http://www.w3.org/1999/02/22-rdf-syntax-ns#>
PREFIX owl:<http://www.w3.org/2002/07/owl#>
PREFIX hoip:<http://purl.bioontology.org/ontology/HOIP/>
PREFIX obo:<http://purl.obolibrary.org/obo/>
SELECT DISTINCT ?sub ?sub_label
WHERE
{
  hoip:HOIP_0060337 rdfs:subClassOf [
                    a owl:Restriction ;
                    owl:onProperty obo:BFO_0000051 ;
                    owl:someValuesFrom ?sub
                    ] .
  ?sub rdfs:subClassOf+ hoip:HOIP_0060021.
  ?sub rdfs:label ?sub_label .
  FILTER (lang(?sub_label) = '')
}
```

**ID**	**Term label**
HOIP_0060337	DNA damage response signaling [adult cellular senescence]
BFO_0000051	has part
HOIP_0060021	cellular senescence course-specific process

2. Find all sub-processes of HOIP_0060324 cellular response to DNA damage stimulus [adult cellular senescence]

```
PREFIX rdfs: <http://www.w3.org/2000/01/rdf-schema#>
PREFIX rdf:<http://www.w3.org/1999/02/22-rdf-syntax-ns#>
PREFIX owl:<http://www.w3.org/2002/07/owl#>
PREFIX hoip:<http://purl.bioontology.org/ontology/HOIP/>
PREFIX obo:<http://purl.obolibrary.org/obo/>
SELECT DISTINCT ?sub ?sub_label ?sub2 ?sub2_label ?sub3 ?sub3_label
WHERE
{
{
  hoip:HOIP_0060324 rdfs:subClassOf [
                    a owl:Restriction ;
                    owl:onProperty obo:BFO_0000051 ;
                    owl:someValuesFrom ?sub
                    ] .
  ?sub rdfs:subClassOf+ hoip:HOIP_0060021.
  ?sub rdfs:label ?sub_label .
  FILTER (lang(?sub_label) = '')
}
OPTIONAL
{
  ?sub rdfs:subClassOf [
                    a owl:Restriction ;
                    owl:onProperty obo:BFO_0000051 ;
                    owl:someValuesFrom ?sub2
                    ] .
  ?sub2 rdfs:subClassOf+ hoip:HOIP_0060021.
  ?sub2 rdfs:label ?sub2_label .
  FILTER (lang(?sub2_label) = '')
}
OPTIONAL
{
  ?sub2 rdfs:subClassOf [
                    a owl:Restriction ;
                    owl:onProperty obo:BFO_0000051 ;
```

```
                        owl:someValuesFrom ?sub3
                        ] .
    ?sub3 rdfs:subClassOf+ hoip:HOIP_0060021.
    ?sub3 rdfs:label ?sub3_label .
    FILTER (lang(?sub3_label) = '')
}
}
```

ID	Term label
HOIP_0060324	cellular response to DNA damage stimulus [adult cellular senescence]
BFO_0000051	has part
HOIP_0060021	cellular senescence course-specific process

3. Find the causal processes of HOIP_0060113 chronic inflammation in chronic cellular senescence course

```
PREFIX rdfs: <http://www.w3.org/2000/01/rdf-schema#>
PREFIX rdf:<http://www.w3.org/1999/02/22-rdf-syntax-ns#>
PREFIX owl:<http://www.w3.org/2002/07/owl#>
PREFIX hoip:<http://purl.bioontology.org/ontology/HOIP/>
PREFIX obo:<http://purl.obolibrary.org/obo/>
SELECT DISTINCT ?process_uri ?process_label ?cause_uri ?cause_label
WHERE
{
  ?process_uri rdfs:label ?process_label .
  FILTER (?process_uri = hoip:HOIP_0060113)
  FILTER (lang(?process_label) = '')
  ?cause_uri rdfs:subClassOf [
                  a owl:Restriction ;
                  owl:onProperty hoip:HOIP_0000069 ;
                  owl:someValuesFrom hoip:HOIP_0060113
                  ] .
  ?cause_uri rdfs:subClassOf+ hoip:HOIP_0060021.
  ?cause_uri rdfs:label ?cause_label .
  FILTER (lang(?cause_label) = '')
}
```

**ID**	**Term label**
HOIP_0060113	chronic inflammation [chronic cellular senescence]
HOIP_0000069	has result
HOIP_0060021	cellular senescence course-specific process

4. Find the molecules with the "SASP factor" role and the processes in which the molecules participate.

```
PREFIX rdfs: <http://www.w3.org/2000/01/rdf-schema#>
PREFIX rdf:<http://www.w3.org/1999/02/22-rdf-syntax-ns#>
PREFIX owl:<http://www.w3.org/2002/07/owl#>
PREFIX hoip:<http://purl.bioontology.org/ontology/HOIP/>
PREFIX obo:<http://purl.obolibrary.org/obo/>
SELECT ?process ?process_label ?mol ?mol_label ?mol_role ?mol_role_label
WHERE {
  ?process rdfs:subClassOf+ hoip:HOIP_0060021.
  ?process rdfs:label ?process_label.
  ?process rdfs:subClassOf [
              a owl:Restriction ;
              owl:onProperty obo:RO_0002234 ;
              owl:someValuesFrom   [
                    owl:intersectionOf (?mol [
                    owl:onProperty obo:RO_0000087 ;
                    owl:someValuesFrom ?mol_role
                    ] )
                ]
  ].
  ?mol rdfs:label ?mol_label.
  ?mol_role rdfs:label ?mol_role_label
   FILTER (?mol_role = hoip:HOIP_0060654) .
  FILTER (lang(?process_label) = '') .
  FILTER (lang(?mol_label) = '') .
    FILTER (lang(?mol_role_label) = '') .
}
```

URI	Term label
HOIP_0060021	cellular senescence course-specific process
RO_0002234	has output
RO_0000087	has role
HOIP_0060654	SASP factor

5. Find the process in which metformin might participate with the "senolytics agent" role and what process may not occur as a result.

```
PREFIX rdfs: <http://www.w3.org/2000/01/rdf-schema#>
PREFIX rdf:<http://www.w3.org/1999/02/22-rdf-syntax-ns#>
PREFIX owl:<http://www.w3.org/2002/07/owl#>
PREFIX hoip:<http://purl.bioontology.org/ontology/HOIP/>
PREFIX obo:<http://purl.obolibrary.org/obo/>
SELECT ?process ?process_label ?mol ?mol_label ?result ?result_label
WHERE
{
{
  ?process rdfs:subClassOf+ hoip:HOIP_0060021.
  ?process rdfs:label ?process_label.
  ?process rdfs:subClassOf [
              a owl:Restriction ;
              owl:onProperty hoip:HOIP_0001868 ;
              owl:someValuesFrom   [
                    owl:intersectionOf (?mol [
                        owl:onProperty obo:RO_0000087 ;
                        owl:someValuesFrom hoip:HOIP_0060420
                        ])
                      ]
                ].
  ?mol rdfs:label ?mol_label.
  FILTER (lang(?process_label) = '') .
  FILTER (?mol =   obo:CHEBI_6801) .
  FILTER (lang(?mol_label) = '') .
}
OPTIONAL
{
  ?process rdfs:subClassOf [
              rdf:type owl:Class ;
              owl:complementOf [
                a owl:Restriction ;
                owl:onProperty hoip:HOIP_0000069 ;
```

```
                        owl:someValuesFrom ?result
                        ]
                  ].
   ?result rdfs:label ?result_label.
   FILTER (lang(?result_label) = '') .
}
}
```

URI	Term label
HOIP_0060021	cellular senescence course-specific process
HOIP_0001868	has agent
RO_0000087	has role
HOIP_0060420	senolytics agent
CHEBI_6801	Metformin
HOIP_0000069	has result

6. Find the direct cause of the processes of HOIP_0060092 p16 signaling (transient) and its causal process in the acute cellular senescence course.

```
PREFIX rdfs: <http://www.w3.org/2000/01/rdf-schema#>
PREFIX rdf:<http://www.w3.org/1999/02/22-rdf-syntax-ns#>
PREFIX owl:<http://www.w3.org/2002/07/owl#>
PREFIX hoip:<http://purl.bioontology.org/ontology/HOIP/>
PREFIX obo:<http://purl.obolibrary.org/obo/>
SELECT
DISTINCT   ?cause2_uri ?cause2_label ?cause_uri ?cause_label ?process_uri ?process_label
WHERE
{
{
  ?process_uri rdfs:label ?process_label .
  FILTER (?process_uri = hoip:HOIP_0060092)
  FILTER (lang(?process_label) = '')
  ?cause_uri rdfs:subClassOf [
                a owl:Restriction ;
                owl:onProperty hoip:HOIP_0000069 ;
                owl:someValuesFrom ?process_uri
                ] .
  ?cause_uri rdfs:subClassOf+ hoip:HOIP_0060021.
  ?cause_uri rdfs:label ?cause_label .
  FILTER (lang(?cause_label) = '')
}
OPTIONAL
{
  ?cause2_uri rdfs:subClassOf [
                a owl:Restriction ;
                owl:onProperty hoip:HOIP_0000069 ;
                owl:someValuesFrom ?cause_uri
                ] .
  ?cause2_uri rdfs:subClassOf+ hoip:HOIP_0060021.
  ?cause2_uri rdfs:label ?cause2_label .
  FILTER (lang(?cause2_label) = '')
}
}
```

ID	Term label
HOIP_0060092	p16 signaling (transient) [acute cellular senescence]
HOIP_0000069	has result
HOIP_0060021	cellular senescence course-specific process

7. Where might SA-beta-GAL accumulation process HOIP_0060626 occur?

```
PREFIX rdfs: <http://www.w3.org/2000/01/rdf-schema#>
PREFIX rdf:<http://www.w3.org/1999/02/22-rdf-syntax-ns#>
PREFIX owl:<http://www.w3.org/2002/07/owl#>
PREFIX hoip:<http://purl.bioontology.org/ontology/HOIP/>
PREFIX obo:<http://purl.obolibrary.org/obo/>
SELECT ?process ?process_label ?location ?location_label
WHERE {
  ?process rdfs:subClassOf+ hoip:HOIP_0060021.
  ?process rdfs:label ?process_label.
  FILTER (?process = hoip:HOIP_0060626)
  FILTER (lang(?process_label) = '')
  ?process rdfs:subClassOf [
                    a owl:Restriction ;
                    owl:onProperty obo:BFO_0000066 ;
                    owl:someValuesFrom ?location
                      ].
  ?location rdfs:label ?location_label.
  FILTER (lang(?location_label) = '') .
}
```

ID	Term label
HOIP_0060626	SA-β-GAL accumulation [chronic cellular senescence]"
BFO_0000066	occurs_in
HOIP_0060021	cellular senescence course-specific process
